# A virtual laboratory based on full-field crystal plasticity simulation to characterize the multiscale mechanical properties of AHSS

**DOI:** 10.1038/s41598-022-09045-8

**Published:** 2022-03-23

**Authors:** Hongyue Ma, Yangqi Li, Haiming Zhang, Qian Li, Fei Chen, Zhenshan Cui

**Affiliations:** 1 School of Materials Science and Engineering, Shanghai Jiao Tong University, 800 Dongchuan Road, Shanghai, 200240 People’s Republic of China; 2 Institute of Forming Technology & Equipment, Shanghai Jiao Tong University, 1954 Huashan Road, Shanghai, 200030 People’s Republic of China; 3 Institute of Shanghai Aircraft Design & Research of Commercial, Aircraft Corporation of China, Jinke Road, Shanghai, 201210 People’s Republic of China

**Keywords:** Theory and computation, Mechanical properties, Metals and alloys

## Abstract

In this work, we proposed a virtual laboratory based on full-field crystal plasticity (CP) simulation to track plastic anisotropy and to calibrate yield functions for multiphase metals. The virtual laboratory, minimally, only requires easily accessible EBSD data for constructing the highly-resolved microstructural representative volume element and macroscopic flow stress data for identifying the micromechanical parameters of constituent phases. An inverse simulation method based on a global optimization scheme was developed to identify the CP parameters, and a nonlinear least-squares method was employed to calibrate yield functions. Mechanical tests of advanced high strength steel sheet under various loading conditions were conducted to validate the virtual laboratory. Three well-known yield functions, the quadratic Hill48 and non-quadratic Yld91 and Yld2004-18p yield functions, were selected as the validation benchmarks. All the studied functions, calibrated by numerous stress points of arbitrary loading conditions, successfully captured both the deformation and strength anisotropies. The full-field CP modeling correlated well the microscopic deformation mechanism and plastic heterogeneity with the macromechanical behavior of the sheet. The proposed virtual laboratory, which is readily extended with physically based CP models, could be a versatile tool to explore and predict the mechanical property and plastic anisotropy of advanced multiphase materials.

## Introduction

During the past decades, advanced lightweight structural metals and alloys, represented by advanced high strength steel (AHSS), are increasingly used to meet the demand for energy conservation and environmental protection^1,2^. The superior mechanical properties and the high strength-to-weight ratio of AHSS are primarily attributed to their synergetic enhancing mechanisms of strength and plasticity through the sophisticated control of the microstructure-property relationship^3^. The complex microstructure, typically consisting of multiple phases over a wide range of length scale, renders their rather different macro-/micro-mechanical responses and plasticity behaviors. Specifically, plastic anisotropy of deformation and strength is one of the most concerns in the community of sheet metal forming^4^.

Describing the plastic anisotropy of metal sheets is a long-standing research focus and challenge. Phenomenological yield functions, which define a convex yield surface to separate the pure elastic and elastoplastic deformation states, are widely employed to predict the macroscopic plastic anisotropy of rate-independent metals for their user-friendly implementation and high computational efficiency. The classic quadratic yield function Hill48^5^, for instance, is the tacit yield criterion for orthotropic materials in most commercial FEM softwares. However, this yield criterion is short of simultaneously capturing deformation anisotropy (mostly measured with *r*-values, i.e., Lankford coefficients) and strength anisotropy^6^. With the emergence of progressive metals and alloys, especially materials with multiphase structure or low-symmetric crystal structure, advanced non-quadratic yield functions have received great attention. Hosford^7^ proposed an isotropic yield criterion without shear stress based on polycrystalline calculation and further extended it to the planar anisotropy^8^. Whereafter, improved yield functions^9–14^ based on linear transformation of stress tensor were introduced to more reasonably describe the anisotropy of materials. Barlat et al.^12^ and Aretz and Barlat^15^ introduced advanced yield functions for general stress state, namely, Yld2004-18p and Yld2004-27p, which require up to 18 and 27 material parameters to describe plastic anisotropy. These yield functions are mostly featured with lots of material parameters and growing alliance with crystallographic texture^9,12^.

The increased number of material parameters causes the tough calibration of the advanced yield functions; the necessary mechanical experiments are time-consuming and sometimes impossible to perform, such as probing the out-of-plane properties of metal sheets. Besides, the phenomenological yield functions are insufficient of physical mechanism and do not consider the microstructural heterogeneity. This weakens their efficiency in predicting the plastic anisotropy of metal sheets with multiphase microstructure, of which the plastic heterogeneity and stress/strain partitions among microstructures and phases should not be ignored^16,17^.

Apart from the phenomenological yield criteria, micromechanical models and simulation tools based on crystal plasticity (CP) theory play a vital role in exploring the plastic anisotropy of metals as well as calibrating advanced yield functions. For instance, Barlat et al.^12^ used a mean-field visco-plastic self-consistent (VPSC) polycrystal plasticity model to evaluate the out-of-plane mechanical property of 2029-T3 Al-Li alloy sheet under different deformation modes; by combining with experimental results, they further identified the complete parameters of the Yld2004-18p yield function for the material. Plunkett et al.^18^ fitted the material parameters of the CPB05 yield function for zirconium by using VPSC simulations.

Recently, full-field CP modeling, either employing finite element method (CPFEM)^19–21^ or spectral method (CPSM)^22,23^ based on fast Fourier transform as the solver of boundary value problems, has received growing interest in characterizing the anisotropic behavior of polycrystalline materials. Full-field CP modeling, taking both grain microstructure and crystallographic texture as input, can guarantee the stress equilibrium and strain compatibility among grains and has the advantage over the mean-field ones in terms of considering the authentic multiphase/polycrystalline microstructures and describing the stress/strain partitions among phases and grains^23,24^. Zhang et al.^25^ employed various CP-based modeling approaches to investigate the anisotropy of AA1050 aluminum and calibrate the anisotropic parameters of the Yld2004-18p yield function. Zhang et al.^23^ proposed a virtual laboratory (VL) based on high-resolution CPSM to predict the yield loci and plastic anisotropy of the annealed and cold-rolled AA3104 aluminum alloy sheets. The material parameters of the Yld91, Yld2000-2D, Yld2004-18p, and Yld2004-27p yield functions were determined through many yield stress data under arbitrary deformation paths. Liu et al.^26^ used the CPSM simulations to calibrate a 3D phenomenological yield function with both in-plane and out-of-plane mechanical anisotropies of strongly textured AA3104-H19 and AA2024-T3 sheets. Han et al.^27^ employed CPSM combined with FEM simulations to dynamically calibrate the Yld2000-2D and Yld1004-18p yield functions for AA2090-T3 sheet at different deformation stages; FEM simulations were used to track the boundary conditions during the deep drawing process. These hierarchical modeling approaches combine the merits of the computational efficiency of phenomenological yield functions and the accuracy of micromechanical CP models.

However, most of the works mentioned above were focused on single-phase materials. This work aims to introduce a full-field CP-based VL capable of characterizing the multiscale mechanical properties of multiphase materials (using DP980 AHSS as an example), including macroscopic mechanical anisotropy and grain-level heterogeneities. Comprehensive experiments were performed for the CP modeling setup and the validation of simulation results. A global optimum inverse simulation procedure was developed to obtain the CP constitutive parameters for the constituent phases of the DP980 sheet. Virtual tests were carried out to obtain adequate stress points of arbitrary loading conditions; these stress points were used to calibrate the exemplified yield functions, i.e., the Hill48, Yld91, and Yld2004-18p, to explore the anisotropy of the DP980 sheet. In the end, the correlation between the grain-level plastic heterogeneity with the macromechanical property of the sheet was investigated.

## Theory

### Finite strain CP theory

For completeness, the adopted phenomenological CP theory is briefly presented; more details can be found in Zhang et al.^28^. The CP model was implemented in a finite strain framework based upon the classical multiplication decomposition of deformation gradient as follows,

1 $$\mathbf{F}={\mathbf{F}}^{\mathbf{e}}{\mathbf{F}}^{\mathbf{p}}$$

where ${\mathbf{F}}^{\mathbf{p}}$ describes the plastic slip on slip planes and ${\mathbf{F}}^{\mathbf{e}}$ describes the elastic stretching and the rotation of lattice. The plastic velocity gradient is expressed as,

2 $${\mathbf{L}}^{{\text{p}}} = \mathop \sum \limits_{\alpha } \dot{\gamma }^{\alpha } {\mathbf{S}}^{\alpha } ,\;{\text{with}}\;\;{\mathbf{S}}^{\alpha } = {\mathbf{m}}^{\alpha } \otimes {\mathbf{n}}^{\alpha }$$

where ${\dot{\gamma }}^{\alpha }$ is the slip rate of the $\alpha$-th slip system, and ${\mathbf{S}}^{\alpha }$ is the Schmid tensor. ${\mathbf{m}}^{\alpha }$ and ${\mathbf{n}}^{\alpha }$ denote the slip direction and the normal of the $\alpha$-th slip system. The slip rate ${\dot{\gamma }}^{\alpha }$ is described by a phenomenological plastic flow law as follows,

3 $$\dot{\gamma }^{\alpha } { = }\dot{\gamma }_{0} \left| {\frac{{\tau^{\alpha } }}{{g^{\alpha } }}} \right|^{1/m} {\text{sign}}\left( {\tau^{\alpha } } \right),\;\;{\text{with}}\;\;\tau^{\alpha } = \left( {{\mathbf{F}}^{{{\text{eT}}}} {\mathbf{F}}^{{\text{e}}} \cdot{\mathbf{T}}^{{\text{e}}} } \right):{\mathbf{S}}^{\alpha }$$

where ${\dot{\gamma }}_{0}$ is the reference shear strain rate, ${\tau }^{\alpha }$ the resolved shear stress acting on the slip system, and ${\mathbf{T}}^{\mathrm{e}}$ the second-order Piola–Kirchhoff stress tensor. $m$ represents the strain rate sensitivity and ${g}^{\alpha }$ is the slip resistance that increases asymptotically from the initial value ${g}_{0}$ to the saturation value ${g}_{\mathrm{s}}$. To represent the work hardening behavior of crystalline materials, the evolution of ${g}^{\alpha }$ is formulated by a rate-form hardening model as follows^29^,

4 $$\dot{g}^{\alpha } = \mathop \sum \limits_{\beta } h_{\alpha \beta } \dot{\gamma }^{\beta } ,\;{\text{with}}\;\;h_{\alpha \beta } = h_{0} \left[ {q + \left( {1 - q} \right)\delta^{\alpha \beta } } \right]\left| {1 - \frac{{g^{\beta } }}{{g_{\infty } }}} \right|^{a}$$

where ${h}_{0}$, ${g}_{\infty }$, and $a$ are material parameters representing the reference self-hardening coefficient, the saturation value of the slip resistance, and the hardening exponent, respectively. Besides, the latent hardening parameter $q$ is routinely assumed to be 1.0 for coplanar slip systems and 1.4 for non-coplanar slip systems^30^.

With the assumption of hypo-elasticity of most metals, ${\mathbf{T}}^{\mathrm{e}}$ can be calculated from the elastic Green strain tensor ${\mathbf{E}}_{\mathrm{e}}$ as

5 $${\mathbf{T}}^{{\text{e}}} = {\mathbb{C}}: {\mathbf{E}}_{{\text{e}}} \;\;{\text{with}}\;\;{\mathbf{E}}_{{\text{e}}} = \frac{1}{2}\left( {{\mathbf{F}}^{{{\text{eT}}}} {\mathbf{F}}^{{\text{e}}} - {\mathbf{I}}} \right)$$

where ${\mathbb{C}}$ is the elasticity tensor; for materials with a cubic crystal structure, ${\mathbb{C}}$ is specified by three parameters, *i.e.*, ${C}_{11}$, ${C}_{12}$, and ${C}_{44}$.

The above CP-based constitutive model in conjunction with an open-source spectral solver DAMASK^31^ enables the VL to carry out a large number of virtual experiments with low computational cost^23^.

### Modeling setup for full-field CP simulations

**Figure 1:**
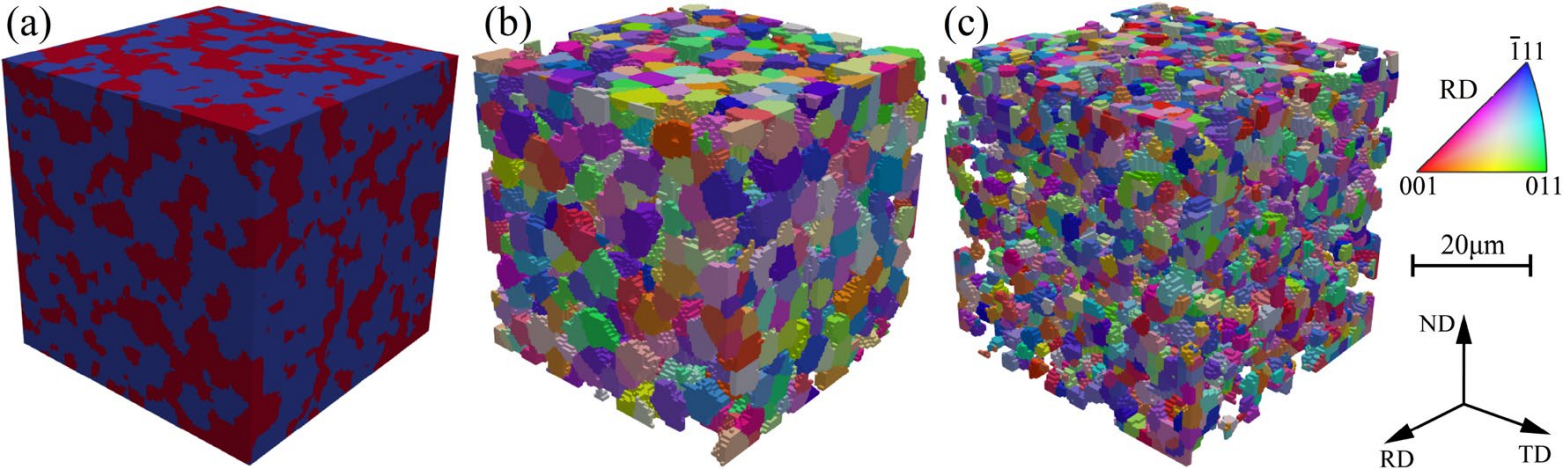
(**a**) The high-resolved dual-phase RVE (DP-RVE) used for CP simulations; red color represents the martensite phase and blue the ferrite. (**b**) and (**c**) are the separated ferrite and (**c**) martensite phases colored with the RD IPF.

The geometrical model used for the full-field CP simulations and virtual tests, i.e., the multiphase microstructural representative volume element (RVE), was constructed via the open-source code DREAM.3D^32^ with the processed EBSD data as input. The cubic RVE, as shown in Fig. 1, contains the essential microstructural characteristics of the DP980 sheet, including the volume fraction, grain size, and ODF of both ferrite and martensite phases. The RVE contains 100 elements (the Fourier grids with grid size of 0.5 μm) in each direction and has 872 orientations (“grains”) in the ferrite phase and 4193 orientations (“islands”) in the martensite, respectively. Periodic boundary conditions were applied to the RVE to perform the simulations. For the simulations of uniaxial tension in different directions from RD to TD, for the convenience of calculating *r*-values, the method of rotating Euler angles of grain orientations instead of alternating the load directions is adopted; more details can be found in Ref.^22^. Following the Bunge convention of Euler angles, the orientation after an in-plane rotation from RD to TD at angle $\theta$ is $\left\{{\varphi }_{1}-\theta ,\right. \Phi ,{\varphi }_{2}$}. In this way, the same RVE and PBCs were used for the full-field CP simulations, and a large number of random stress states were simulated for calibrating the phenomenological yield functions.

### Parameter identification for the CP model

The dual-phase steel has a composite microstructure of relatively strong martensite islands dispersed in the soft and ductile ferrite matrix. Both the ferrite and the martensite phases have a BCC crystal structure (herein, we ignore that slight distortion of the martensite’s structure from the BCC structure) with the most easily activated slip system families $\left\{\bar{1}10\right\}\langle 111\rangle$ and $\left\{\bar{2}11\right\}\langle 111\rangle$ at room temperature; each slip system family has an individual set of parameters. Thus, there are a lot of material parameters to be identified. To identify the mechanical properties of individual phases in AHSS, Hu et al.^33,34^ employed the state-of-the-art high-energy X-ray diffraction technique and in-situ tensile tests to measure the lattice strain of each phase; they calibrated the CP parameters by correlating the predicted lattice strains with the experiments. This approach is attractive and persuasive but not easily accessible. In this work, an inverse simulation method evolving global optimization was developed to identify the CP constitutive parameters of the ferrite and martensite phases. First, the elastic constants ${C}_{11}$, ${C}_{12}$, and ${C}_{44}$, the reference slip rates ${\dot{\gamma }}_{0}$, and the rate sensitivity coefficients $m$ were assumed identical for the two slip systems of each phase, and their values were routinely documented^35^. It is noted that the elastic properties of the constituent phases in multiphase steels are not fully understood. Souissi and Numakura^36^ gave a comprehensive study on the elastic properties of two types of martensite. The four parameters, ${g}_{0}$, ${g}_{\infty }$, ${h}_{0}$, and $a$, which describe the work hardening behavior of slip systems, were identified by an in-house inverse simulation procedure based on the uniaxial tensile data in RD and TD. According to the research of Tasan et al.^35^ and Hamada et al.^37^, the ratio of ${g}_{0}$ in the $\left\{\bar{1}10\right\}\langle 111\rangle$ and $\left\{\bar{2}11\right\}\langle 111\rangle$ slip systems is approximately 1.0 ~ 1.1. Thus, a constraint was enforced to make the initial and saturation slip resistances ${g}_{0}$ and ${g}_{\infty }$ of system $\left\{\bar{1}10\right\}\langle 111\rangle$ smaller than those of $\left\{\bar{2}11\right\}\langle 111\rangle$.

**Figure 2:**
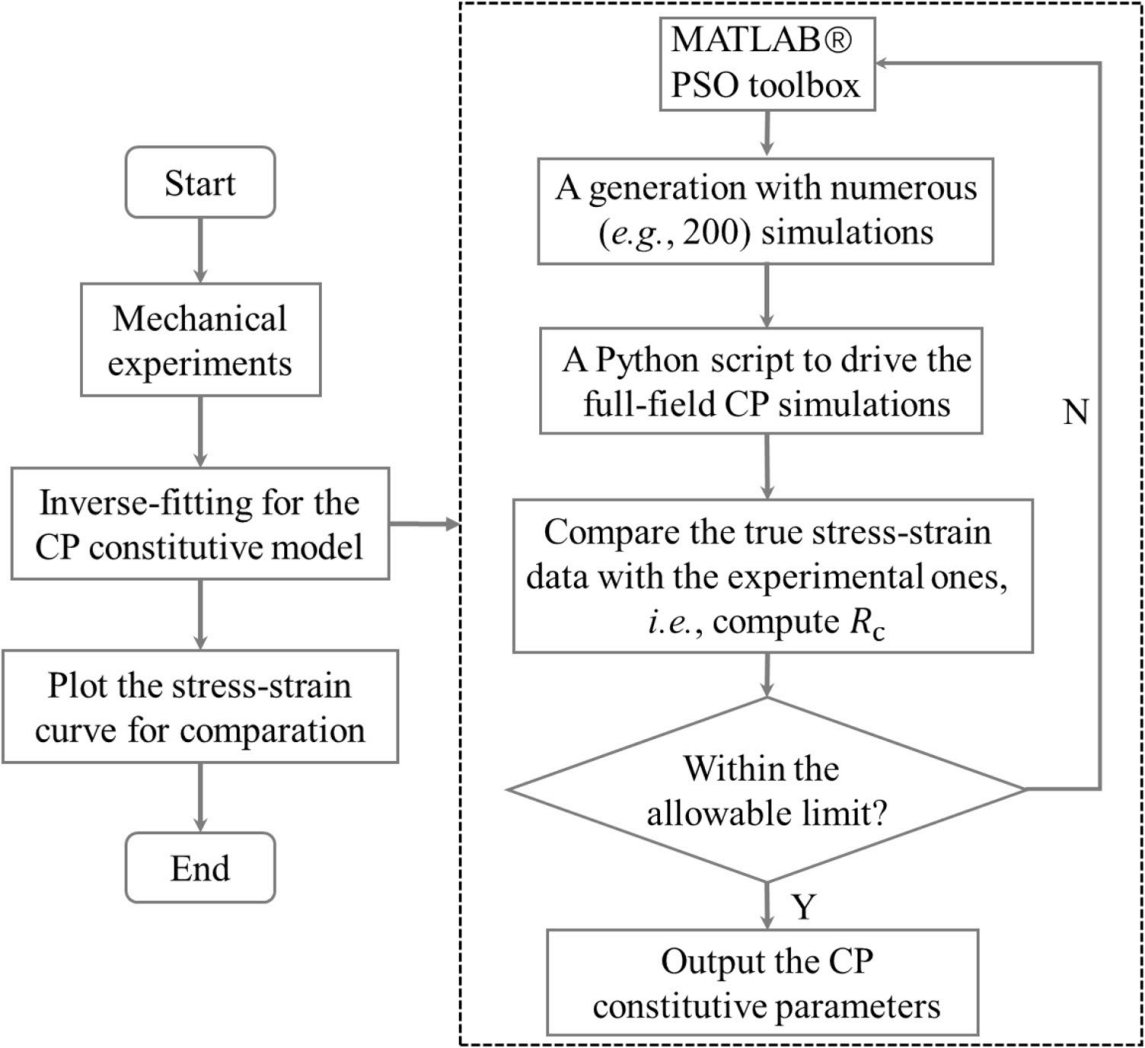
The flow chart of the in-house inverse simulation method for identifying the CP model parameters.

The inverse simulation method for parameter identification consists of a global optimization platform and full-field CP simulations. The particle swarm optimization (PSO) toolbox of MATLAB® (version R2018b) was employed to identify the global optimum material parameters for the ferrite and martensite phases. In the beginning, reasonable bounds of the parameters were provided for the inverse simulation platform. The platform generated different initial guesses for the parameters, launched numerous full-field CP simulations of uniaxial tension parallelly, and finally extracted the predicted true stress–strain data. The obtained true stress–strain data were compared with the experiment ones to evaluate the root-mean-square deviations (RMSD) ${R}_{\mathrm{c}}$ of the objective function in the current generation. Then the PSO toolbox introduced a new generation with optimized parameter sets. The fitting process will be iterated until the minimum ${R}_{\mathrm{c}}$ below the prescribed limit. Figure 2 shows the flow chart of the inverse simulation procedure for identifying the CP constitutive parameters. The objective function of the PSO optimization procedure reads,

6 $${R}_{\mathrm{c}}=\sum_{i=1}^{n}{w}_{i}{\left[\left({\sigma }_{i}^{\mathrm{sim}}\left(x\right)-{\sigma }_{i}^{\mathrm{exp}}\right)/{\sigma }_{i}^{\mathrm{exp}}\right]}^{2}$$

where $x=\left[{x}_{1},{x}_{2},{\dots ,x}_{n}\right]$ are the parameters to be identified. ${\sigma }_{i}^{\mathrm{sim}}$ and ${\sigma }_{i}^{\mathrm{exp}}$ are the simulated and experimental stresses at the same strain, respectively, and $i$ denotes the corresponding data in different directions, i.e., RD and TD. ${w}_{i}$ is the weighting factor, which is set to 1.0 in this work.

**Figure 3:**
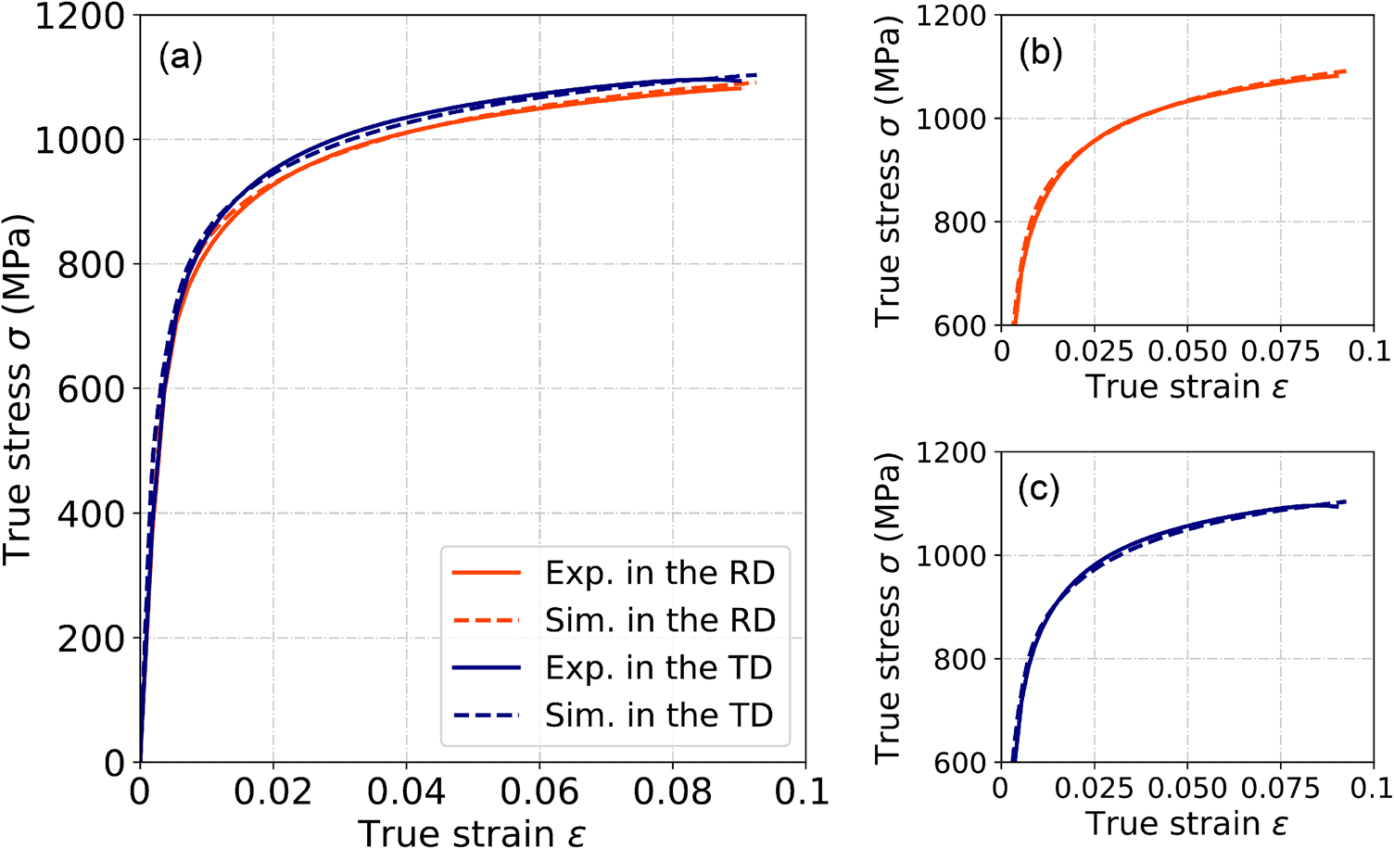
(**a**) Comparison of the flow stress curves predicted the calibrated CP model with the experimental ones along RD and TD. The partial magnifications of the results in RD and TD are shown in subfigures (**b**) and (**c**), respectively.

Table 1 lists all the CP constitutive parameters used in this work. Figure 3 shows the experimental and the CP simulated flow stress curves of uniaxial tension in both RD and TD. For ${g}_{0}$, the values of two slip systems of $\left\{\bar{1}10\right\}\langle 111\rangle$ and $\left\{\bar{2}11\right\}\langle 111\rangle$ in the martensite are 4 ~ 5 times higher than those in the ferrite; for ${g}_{\infty }$, the values of $\left\{\bar{1}10\right\}\langle 111\rangle$ and $\left\{\bar{2}11\right\}\langle 111\rangle$ in the martensite are 3 ~ 4 and 1.5 ~ 2.5 times higher than those in the ferrite, respectively.

**Table 1:** Material parameters adjusted to the mechanical properties of both ferrite and martensite phases.

Parameters	Ferrite		Martensite		Unit
	$\left\{\bar{1}10\right\}\langle 111\rangle$	$\left\{\bar{2}11\right\}\langle 111\rangle$	$\left\{\bar{1}10\right\}\langle 111\rangle$	$\left\{\bar{2}11\right\}\langle 111\rangle$	
${g}_{0}$	180.7	216.9	800.0	941.7	MPa
${g}_{\infty }$	305.9	441.6	980.0	980.0	MPa
${h}_{0}$	1.8	1.9	1.0	1.0	GPa
$a$	1.05	1.40	2.18	1.05	–
${\dot{\gamma }}_{0}$	0.001		0.001		s^−1^
*m*	0.02		0.02		–
${C}_{11}$	233.3		417.4		GPa
${C}_{12}$	135.5		242.4		GPa
${C}_{44}$	118.0		211.1		GPa

Parameters of the initial and saturation slip resistances, reference self-hardening modulus, and hardening exponent were determined by an in-house inverse simulation procedure based on the experimental data; other parameters were routinely documented and referred to the literature^35^.

### Virtual laboratory

The VL is executed automatically with a Python script. It launches enough full-field CP simulations of the constructed RVE under arbitrary loading conditions generated by a random number generator, post-processes the simulation results, extracts the yield stress points (stress tensors), and then identifies the yield functions. As the illustrative benchmarks, three well-known yield functions, i.e., the quadratic Hill48^5^ and non-quadratic Yld91^9^ and Yld2004-18p^12^ yield functions, were calibrated to predict the anisotropy of the DP980 sheet.

The nonlinear least-squares method (NLSM) was adopted to identify the coefficients of the studied yield functions. The NLSM problem is solved by using a bounded Levenberg–Marquardt optimization algorithm^23^. With the analytical Jacobian matrix, this algorithm provides enough robustness to find the solution of strong-nonlinear issues, even though the initial guess starts far off the final minimum. The objective function $O\left(\beta \right)$ is defined as follows.

7 $$O\left(\beta \right)={\sum }_{i=1}^{N}{\left(\frac{\phi \left({\sigma }^{i},{\beta }_{j}\right)}{{\sigma }_{y}}-1\right)}^{2}$$

$\phi \left({\sigma }^{i},{\beta }_{j}\right)$ denotes the effective stress calculated by the calibrated yield functions, and ${\sigma }^{i} \left(i=\mathrm{1,2},\dots ,N\right)$ are the set of stress tensors of the corresponded virtual tests at the same plastic work per unit volume (shorten as the specific plastic work in the following content), and ${\beta }_{j}={\beta }_{1},{\beta }_{2},\dots ,{\beta }_{M}$ are the parameters of the yield functions to be calibrated. The optimal parameters $\left\{{\beta }_{j}\right\}$ minimize the objective function $O\left(\beta \right)$. All the stress tensors were assigned with the same unit weight in this study; however, different weights can be endowed to realize the strong dependence of certain stress states. To stabilize and to accelerate the solving process of the objective function $O\left(\beta \right)$, analytical Jacobian matrices were derived for the yield functions; the form of the Jacobian matrices reads as follows,

8 $$J_{ij} = \frac{1}{{\sigma_{y} }}\frac{{\partial \phi \left( {\sigma^{i} ,\beta } \right)}}{{\partial \beta_{j} }},{\text{ with}}\;\;1 \le i \le N\;\;{\text{and}}\;\;1 \le j \le M,$$

with $N$ and $M$ denote the number of stress points and the number of material parameters, respectively. For complex yield functions, e.g., the Yld2004-18p, the chain rule was employed to get the Jacobian matrices. More details can be found in the Supplementary.

The fitting quality is measured via RMSD (${R}_{\mathrm{y}}$) of the residual as follows,

9 $${R}_{\mathrm{y}}= \sqrt{\frac{1}{n}\sum_{n=1}^{N}{\left(\frac{\phi \left({\sigma }^{i},{\beta }_{j}\right)}{{\sigma }_{y}}-1\right)}^{2}}$$

Apart from the VL, which was used to generate the random stress points, full-field CP simulations of uniaxial tension at a ${7.5}^{\mathrm{o}}$ interval from RD to TD were also executed to predict the in-plane anisotropy of the DP980 sheet, and the same RVE was used. Finally, $r-$values (the Lankford coefficients) and normalized yield stresses were extracted from the results of the CP simulations.

## Results and discussion

### Microstructure

The studied material is a commercial cold-rolled and annealing DP980 steel sheet with a thickness of 1.2 mm provided by Baosteel. The chemical composition of the steel is summarized in Table S1 in the Supplementary.

**Figure 4:**
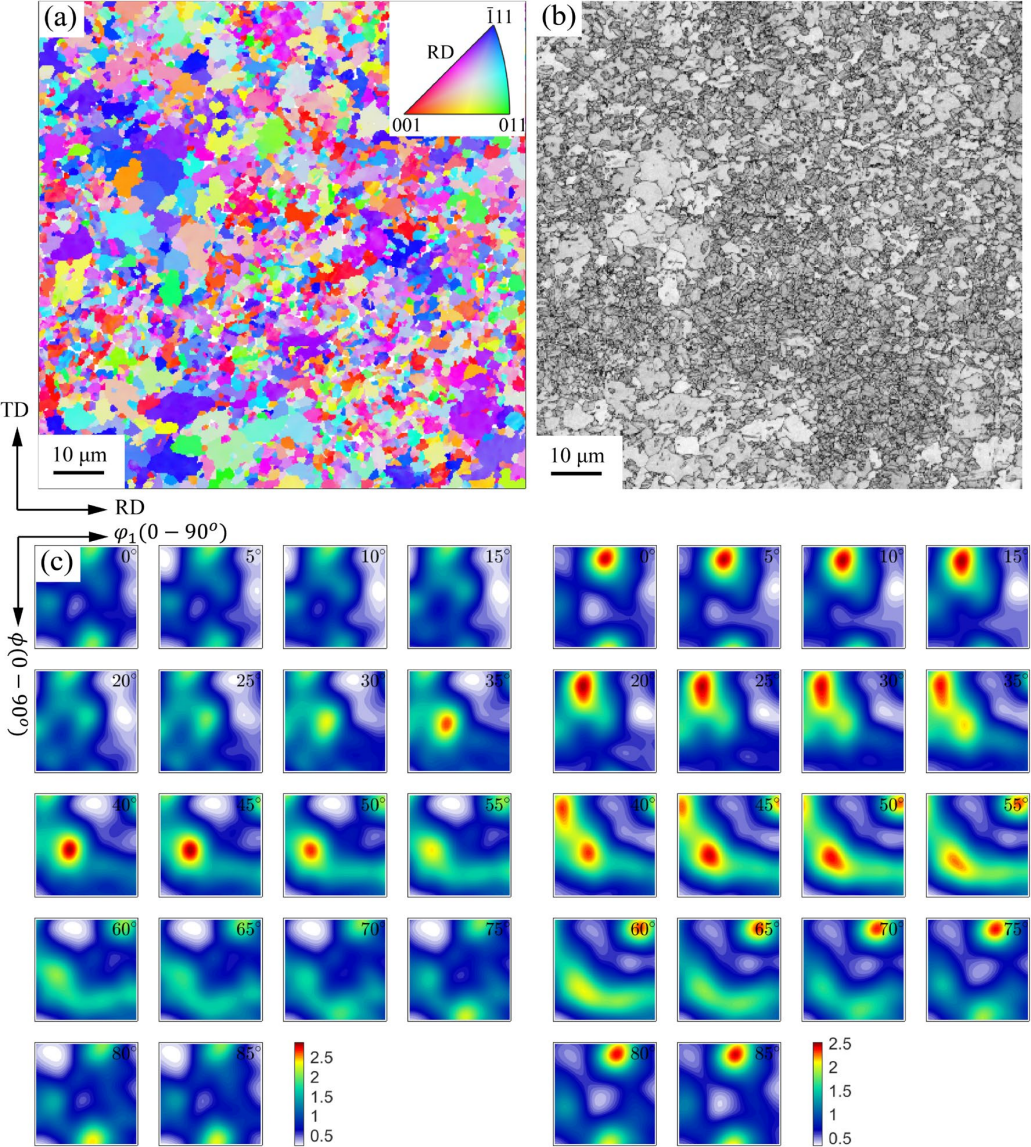
The initial microstructure of the as-received DP980 sheet characterized on the RD-TD plane at the center in thickness. (**a**) The EBSD orientation imaging map (OIM); (**b**) the band contrast map; (**c**) the orientation density function (ODF) maps reconstructed from the EBSD data of the ferrite and martensite phases, respectively. The ODFs were sectioned (constant ${\varphi }_{2}$ sections, from ${0}^{\mathrm{o}}$ to ${85}^{\mathrm{o}}$ in ${5}^{\mathrm{o}}$ steps) through the reduced Euler space (with the Bunge convention) for the cubic-orthorhombic symmetry. This figure was generated by the MATLAB® (version R2018b) open-source toolbox MTEX^38^.

Figure 4 displays the microstructure of the material characterized on the RD–TD plane in the center of thickness. Figure 4a is the orientation imaging map (OIM) obtained from EBSD data, and the OIM is colored with the RD inverse pole figure (IPF). Based on the band contrast value, as shown in Fig. 4b, the original microstructure was separated into the individual ferrite and martensite phases^17^. Figure 4c presents the orientation distribution function (ODF) maps reconstructed from the EBSD data of two individual phases. Both phases show the typical texture of BCC metals after cold-rolling and annealing operations, i.e., the *γ*-fiber consisting of $\left\{111\right\}\langle uvw\rangle$ orientations and the *α*-fiber consisting of $\left\{hkl\right\}\langle 110\rangle$. However, the dominant texture components in the ferrite phase are different from those in the martensite phase. For the ferrite, the intensity of *α*-fiber is quite smaller than that of *γ*-fiber, and *γ*-fiber indeed concentrates at the texture component of $\left\{111\right\}\langle 1\bar{2 }1\rangle$; for the martensite, on the contrary, the intensity of *α*-fiber is comparable with *γ*-fiber, and *α*-fiber concentrates at $\left\{001\right\}\langle 110\rangle$ and the *γ*-fiber at $\left\{111\right\}\langle 1\bar{2 }1\rangle$. It suggests sufficient annealing of the ferrite phase, as the annealing process of cold-rolled BCC materials strengthens *γ*-fiber and reduces the intensity of *α*-fiber^22^.

### The flow stress directionality and yield loci of the DP980 sheet

**Figure 5:**
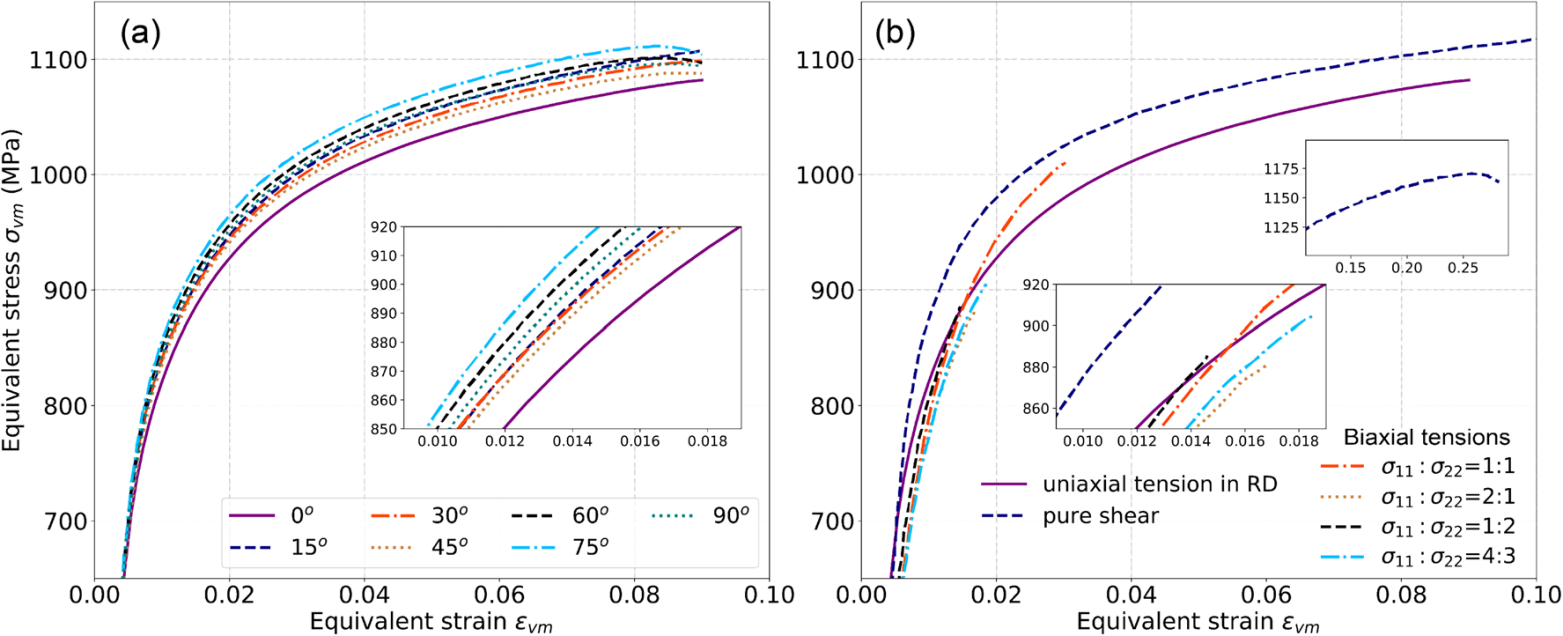
Flow stress curves of (**a**) uniaxial tension (UT) in different directions and (**b**) pure shear and biaxial tension with different stress ratios. ${\sigma }_{11}:{\sigma }_{22}$ represents the stress ratio of the normal stresses in RD and TD.

Figure 5 presents the flow stress curves of the DP980 sheet subjected to different loading conditions. Figure 5a shows the curves of uniaxial tension in different directions, and Fig. 5b the curves of pure shear and biaxial tensile tests. The results exhibit the typical characteristics of cold-rolled dual-phase steels, i.e., no obvious yield point and no stress plateau but continuous hardening. The von Mises equivalent stress of pure shear is higher than the flow stresses of uniaxial tension in RD and biaxial tension with different stress ratios, and the biaxial tensile flow stresses are a little smaller than that uniaxial tensile one in the early deformation stage. With the increase of deformation, the equibiaxial flow stress is higher than the uniaxial tensile stress.

**Figure 6:**
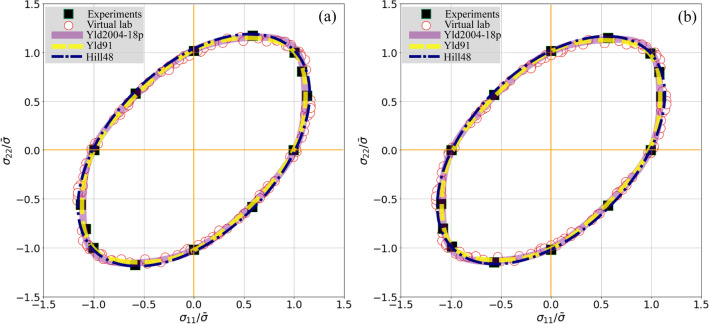
Yield loci $\left({\sigma }_{12}=0\right)$ of the DP980 sheet outlined by the experimental stress points (solid squares) of different loading conditions, enclosed by 60 random stress points (open circles) generated by the VL, and predicted by the calibrated yield functions (lines); the equivalent deformation stage with the specific plastic work of 3 MPa (**a**) and 8 MPa (**b**). $\bar{\sigma }$ denotes the uniaxial tensile stress in RD obtained from the virtual tests.

Figure 6 presents the 2D yield loci of the calibrated yield functions in the ${\sigma }_{11}-{\sigma }_{22}$ space, 60 stress points (open circles) randomly generated by the VL, and experimental results (solid squares) of uniaxial tension in RD and TD, pure shear, and biaxial tension with different stress ratios. For the studied DP980 sheet, the equivalent equibiaxial tensile stress is almost identical with the uniaxial tensile stress in RD but slightly higher than that in TD. The VL simulated and experimental stress points were selected from the deformation stages with the specific plastic work of 3 MPa (Fig. 6a) and 8 MPa (Fig. 6b). All the stress points were normalized by the corresponding uniaxial tensile stress in RD. To verify the VL’s capacity in predicting the plastic anisotropy of multiphase materials, only the simulated 60 stress points and no *r*-values were used to calibrate the yield functions. Table 2 lists the identified parameters of these functions. Different from the recommendation of Hosford^7^, i.e., $m=8$ for materials with FCC crystal structure and $m=6$ for BCC structure, the intentionally calibrated homogeneous exponent $m$ varies in the range of 5 ~ 8.

**Table 2:** The optimal parameters of the calibrated Hill48, Yld91, and Yld2004-18p yield functions for the DP980 sheet.

Yield functions	Specific plastic work	Identified parameters						
Hill48		$F$	$G$	$H$	$L$	$M$	$N$	
	3 MPa	0.4809	0.5364	0.4822	1.5120	1.4272	1.5985	
	8 MPa	0.4509	0.5029	0.4548	1.4035	1.3431	1.4958	
Yld91		$a$	$b$	$c$	$f$	$g$	$h$	$m$
	3 MPa	0.9852	1.0455	1.0030	0.9942	0.9848	1.0391	5.5246
	8 MPa	0.9519	1.0153	0.9680	0.9608	0.9537	1.0070	5.7713
Yld2004-18p		${c}_{12}$	${c}_{21}$	${c}_{23}$	${c}_{32}$	${c}_{31}$	${c}_{13}$	${c}_{44}$
	3 MPa	0.9530	1.0290	0.9299	0.9317	1.1268	1.0910	0.9963
	8 MPa	0.7263	1.1876	1.1016	0.8761	1.1304	0.9469	0.9776
		${c}_{55}$	${c}_{66}$	${d}_{12}$	${d}_{21}$	${d}_{23}$	${d}_{32}$	${d}_{31}$
	3 MPa	0.0292	1.1575	0.9340	0.8417	0.9290	0.9620	0.6287
	8 MPa	0.5326	1.2619	0.8406	0.9662	0.9852	0.8030	0.6843
		${d}_{13}$	${d}_{44}$	${d}_{55}$	${d}_{66}$	$m$		
	3 MPa	0.9612	0.9853	1.4402	0.7265	7.2904		
	8 MPa	1.0006	0.9917	1.3642	0.5933	6.8343		

As shown in Fig. 6, the yield loci predicted by the VL (the randomly distributed stress points represented by the open circles) agree well with the experimental one (enclosed by the solid squares), i.e., the VL successfully reproduced the yield loci of the DP980 sheet. All the calibrated yield functions finely outline the yield loci of the sheet in comparison with both the experimental and the VL results. In particular, the yield loci predicted by the Yld91 and Yld2004-18p functions are quite coincident. In contrast, those predicted by the Hill48 yield function show a large deviation from others in the regions close to equibiaxial tension and plane strain. This is because the Hill48 yield function is insufficient in capturing the large curvature change of yield locus in the region close to equibiaxial tension.

**Figure 7:**
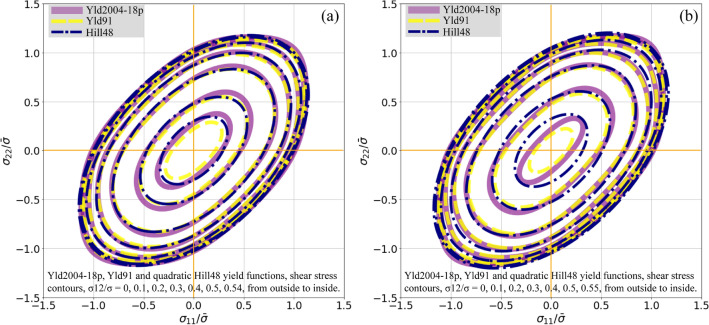
Tricomponent (${\sigma }_{11}-{\sigma }_{22}-{\sigma }_{12}$) yield loci with constant ${\sigma }_{12}/\bar{\sigma }$ contours predicted by the calibrated yield functions for the DP980 sheet. The equivalent deformation stage with the specific plastic work of (**a**) 3 MPa and (**b**) 8 MPa. $\bar{\sigma }$ denotes the uniaxial tensile stress in RD obtained from the VL.

Figure 7 shows a direct comparison of yield loci of all the considered yield functions at equal normalized shear stress ${\sigma }_{12}/\bar{\sigma }$. The maximum ${\sigma }_{12}/\bar{\sigma }$ of the Yld2004-18p, Yld91, and Hill48 yield functions are 0.56009, 0.54134, and 0.56488 at the specific plastic work of 3 MPa, and 0.56057, 0.55641, and 0.58394 at the specific plastic work of 8 MPa, respectively. It can be seen from the results that the deviation among the yield functions increases with the ratio of shear stress, and the difference is noticeable in the case of ${\sigma }_{12}/\bar{\sigma }>0.5.$

### In-plane anisotropy of deformation and strength

**Figure 8:**
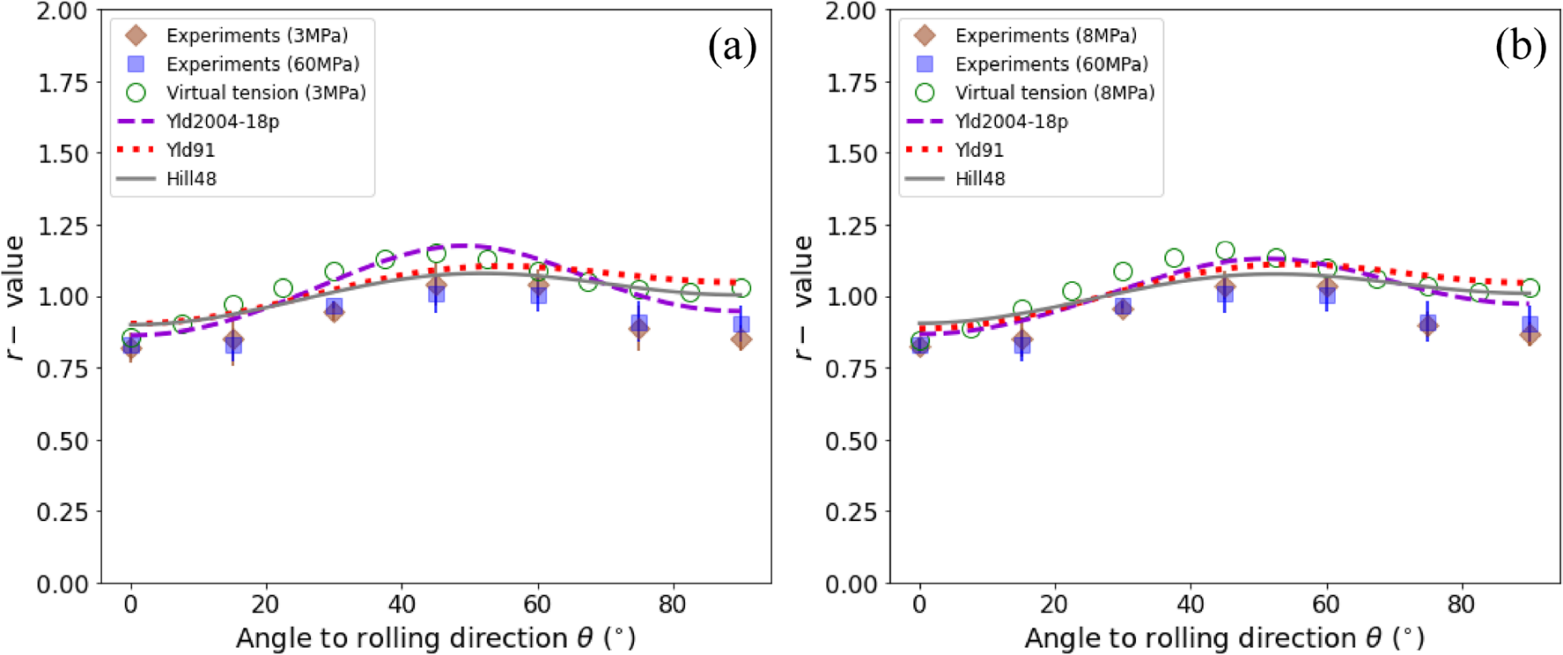
*r*-value versus $\theta$ of the DP980 sheet at deformation stages with the specific plastic work of (**a**) 3 MPa and (**b**) 8 MPa. The lines are the predicted results of the calibrated yield functions. The diamonds and squares represent experimental values at an interval of 15° from RD to TD and the open circles are the results obtained from virtual tests of uniaxial tension at an interval of 7.5° from RD to TD.

**Figure 9:**
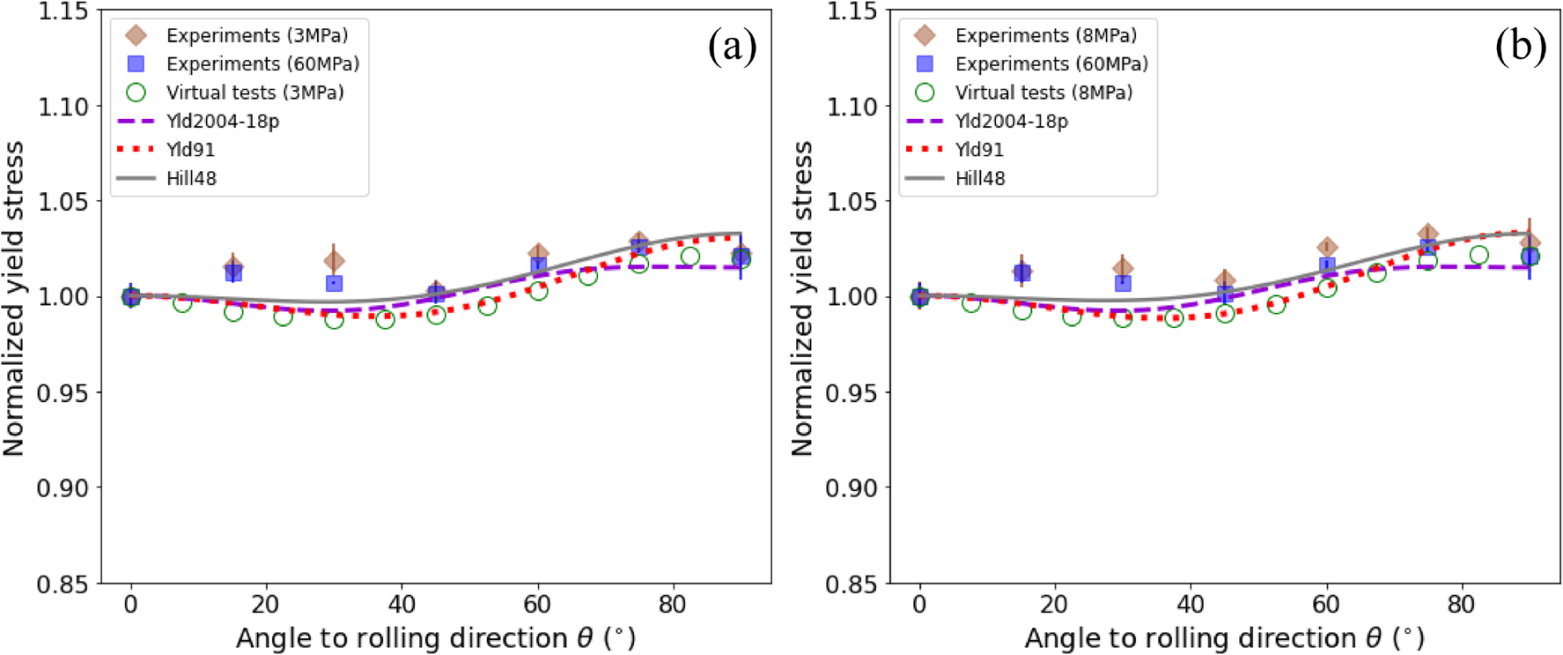
Normalized yield stress ${Y}_{\theta }$ versus $\theta$ of the DP980 sheet at deformation stages with the specific plastic work of (**a**) 3 MPa and (**b**) 8 MPa. The lines are the predicted results of the calibrated yield functions. The diamonds and squares represent experimental values at an interval of 15° from RD to TD and the open circles are the results obtained from virtual tests of uniaxial tension at an interval of 7.5° from RD to TD.

For polycrystalline metal sheets, the variations of *r*-value and normalized yield stress (${Y}_{\theta }$) with uniaxial tensile directions are commonly employed to measure the in-plane anisotropy of deformation and strength. Figure 8 presents the comparisons of the *r*-values predicted by the calibrated yield functions and obtained from the experiments and the virtual tests of uniaxial tension. Figure 8a and b correspond to the results at deformation stages with the specific plastic work of 3 MPa and 8 MPa, respectively. Figure 9 presents the normalized yield stresses. Note that the experimental results at the specific plastic work of 60 MPa were also provided for checking the variation of *r*-values with deformation.

As shown in Fig. 8, the *r*-values obtained from the experiments are fairly consistent with those predicted by the virtual tests and by the calibrated yield functions. The DP980 sheet exhibits the typical deformation anisotropy of most cold-rolled and annealing BCC metal sheets, i.e., the maximum *r*-value in the diagonal direction (DD, i.e., the uniaxial tensile direction aligned 45° with respect to the RD) and the minimum in both RD and TD. The sheet has a small normal anisotropy with the average $\bar{r }$ ($=\left({r}_{0}+{2r}_{45}+{r}_{90}\right)/4$) about 0.941 but a relatively strong in-plane anisotropy with $\Delta r/\bar{r }$ ($\Delta r=2{r}_{45}-{r}_{0}-{r}_{90}$) about 0.418. The full-field CP virtual tests captured the variation tendency of *r*-values with the direction angle $\theta$ very well. Nevertheless, it is noticed that the *r*-values obtained by the virtual tests are a little higher than the experiments; this is might because the present CP model did not consider the potential $\langle 111\rangle$ pencil-glide mechanism of BCC metals^39^, which does not prescribe a specific slip plane for the $\langle 111\rangle$ dislocation glide and thus enables a large number of alternative slip systems. As also stated by Yazawa et al.^40^, limited slip systems increase plastic deformation anisotropy, which is advantageous for realizing higher *r*-values for BCC steels. In other words, a more advanced physically-based CP might be able to yield an even better prediction of the plastic anisotropy for this type of AHSS. In addition, the experimental data evidence that the variation of *r*-values with deformation is not noticeable. At the deformation stages with the specific plastic work of 3 MPa and 8 MPa, the *r*-values are hardly distinguishable in terms of deformation; with the deformation increased to 60 MPa, a small decrease of $\bar{r }$ can be deduced from Fig. 8, i.e., the decrease of *r*-values in the directions near DD and the increase of *r*-values in both RD and TD.

The yield functions, as stated, calibrated with the VL generated stress points only, successfully captured the variation tendency of *r*-values. Especially, the advanced yield function Yld2004-18p predicts the variation of *r*-values in a rather good agreement with both the virtual tests and experimental results; other two yield functions, i.e., Hill48 and Yld91, slightly underestimate $\Delta r$ or the in-plane anisotropy. This is expectable as both have fewer material parameters for describing plastic anisotropy. The Yld91 criterion is a subset version of the Yld2004-18p; the former shares only one-half (either the parameter matrix $\mathbf{C}$ or $\mathbf{D}$ illustrated in Supplementary Eqs. 16–17 of the parameters of the latter. The quadratic Hill48 yield function also predicts a faithful directionality of the *r*-values; this is, to some extent, attributed to the plain variation of the ${r}_{\theta }$ curve. Even though the studied DP980 sheet consists of a dual-phase microstructure, it has only two stationary points in the ${r}_{\theta }$ curve; one locates in the RD/TD and another near the DD. The quadratic Hill48 criterion, as anticipated, is capable of capturing this phenomenon. In a word, although only the VL generated stress points were used to identify the anisotropy parameters of the yield functions, all the calibrated functions successfully capture the directionality and variation of *r*-values. The discrepancies among the predicted results of these functions can also be associated with the intrinsic characteristics of these functions.

As shown in Fig. 9, the DP980 sheet exhibits a small strength anisotropy with the maximum variation of the normalized yield stress ${Y}_{\theta }$ below 5%; this can also be demonstrated by the flow stress curves shown in Fig. 5. With the increase of deformation, the strength anisotropy was slightly weakened. Regardless of the rather small variation of ${Y}_{\theta }$, the stress points obtained by the virtual tests agree well with the experimental data except those in the directions of $\theta ={30}^{\mathrm{o}}$ and ${45}^{\mathrm{o}}$. All the calibrated yield functions predict a well consistent variation of ${Y}_{\theta }$ with the VL results.

It is noted that the Hill48 yield function, which is well-known for its incapacity of capturing the deformation anisotropy and the strength anisotropy simultaneously, gives a satisfactory prediction of the variations of both *r*-values and normalized yield stresses with tensile direction. Because of the cost and sometimes the restriction of physical experiments, the conventional calibration process of yield functions employs the equal-number experimental data with the parameter number to identify the anisotropic parameters. As stated by Zhang et al.^23^, this routine cannot yield an optimum set of parameters. The Hill48 function, for instance, may predict the deformation anisotropy well but the strength anisotropy poorly when calibrated by the experimental data of *r*-values, and vice versa. By using enough number (60 here) of stress points with arbitrary loading conditions, the studied yield functions, including the classic quadratic function and advanced non-quadratic functions, calibrated by the full-field CP-based VL, can well predict the deformation and strength anisotropies simultaneously.

### Plasticity heterogeneity at the grain level

**Figure 10:**
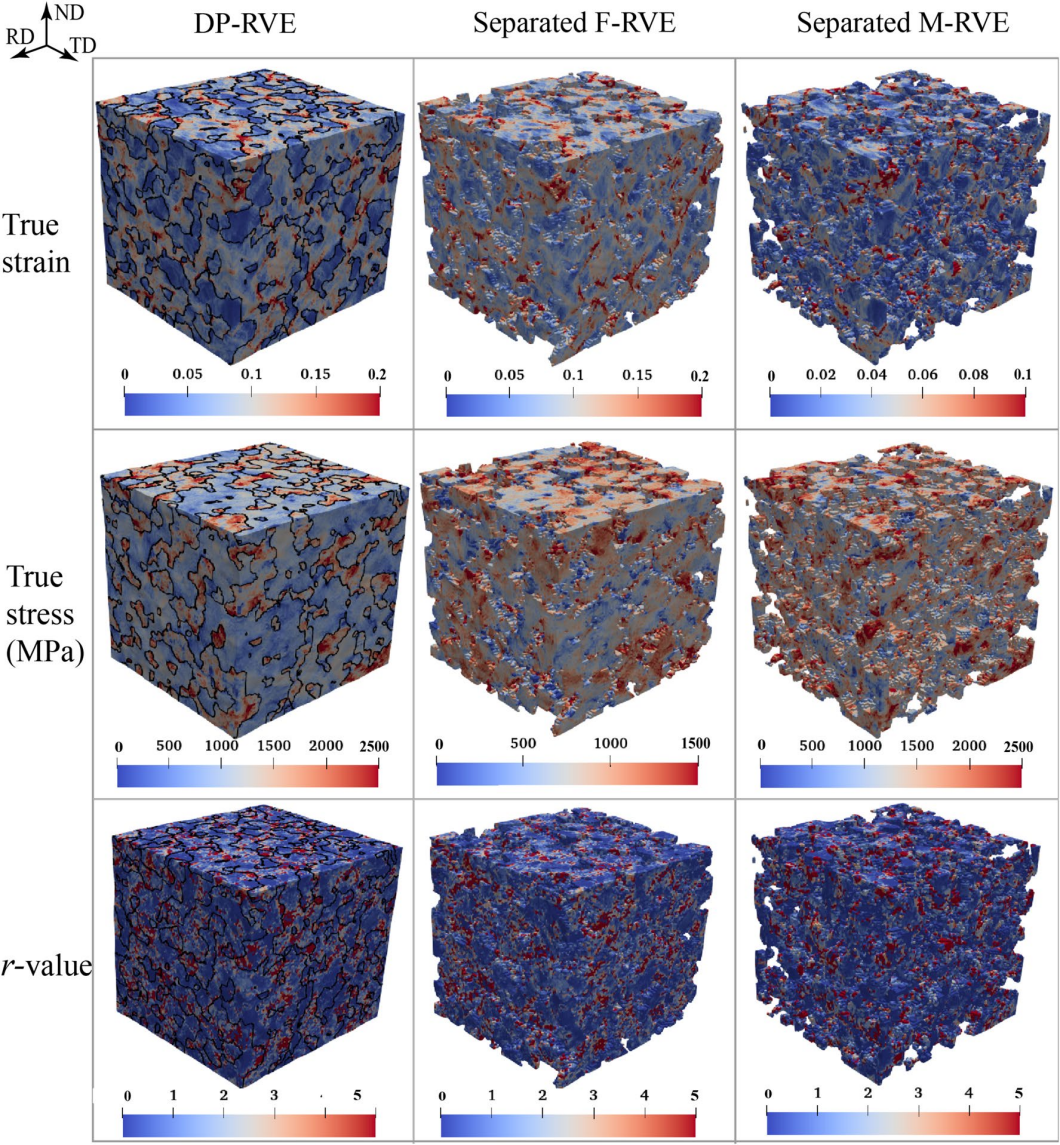
The contour maps of the true strain, true stress, and *r*-value of the DP-RVE and the separated M-RVE and F-RVE after the uniaxial tension in RD to the specific plastic work of 60 MPa. The true stress and true strain correspond to the components in the tensile direction.

To study the role of individual phases in affecting the overall plasticity of the sheet, the microscopic distributions of tensile stress, tensile strain, and *r*-value were further investigated. Figure 10 presents the contour maps of the deformed RVEs tensile tested in RD at the specific plastic work of 60 MPa; both the as-tested dual-phase RVE (DP-RVE) and the intentionally separated single-phase RVEs (F-RVE for ferrite and M-RVE for martensite) were presented for comparison. The black lines in the DP-RVE depict the phase boundaries. As anticipated, all the RVEs exhibit significantly inhomogeneous distributions of stress, strain, and *r*-value. The martensite phase, for its higher strength, shows a larger stress level and a smaller strain level in comparison with the ferrite phase. In contrast, the distributions of *r*-value in the two phases are quite similar and strongly inhomogeneous. The contour maps of the DP-RVE manifest that strain hot-spots occur in some grains’ interior and phase boundaries in the ferrite phase, whereas stress hot-spots are mainly in the martensitic grains.

**Figure 11:**
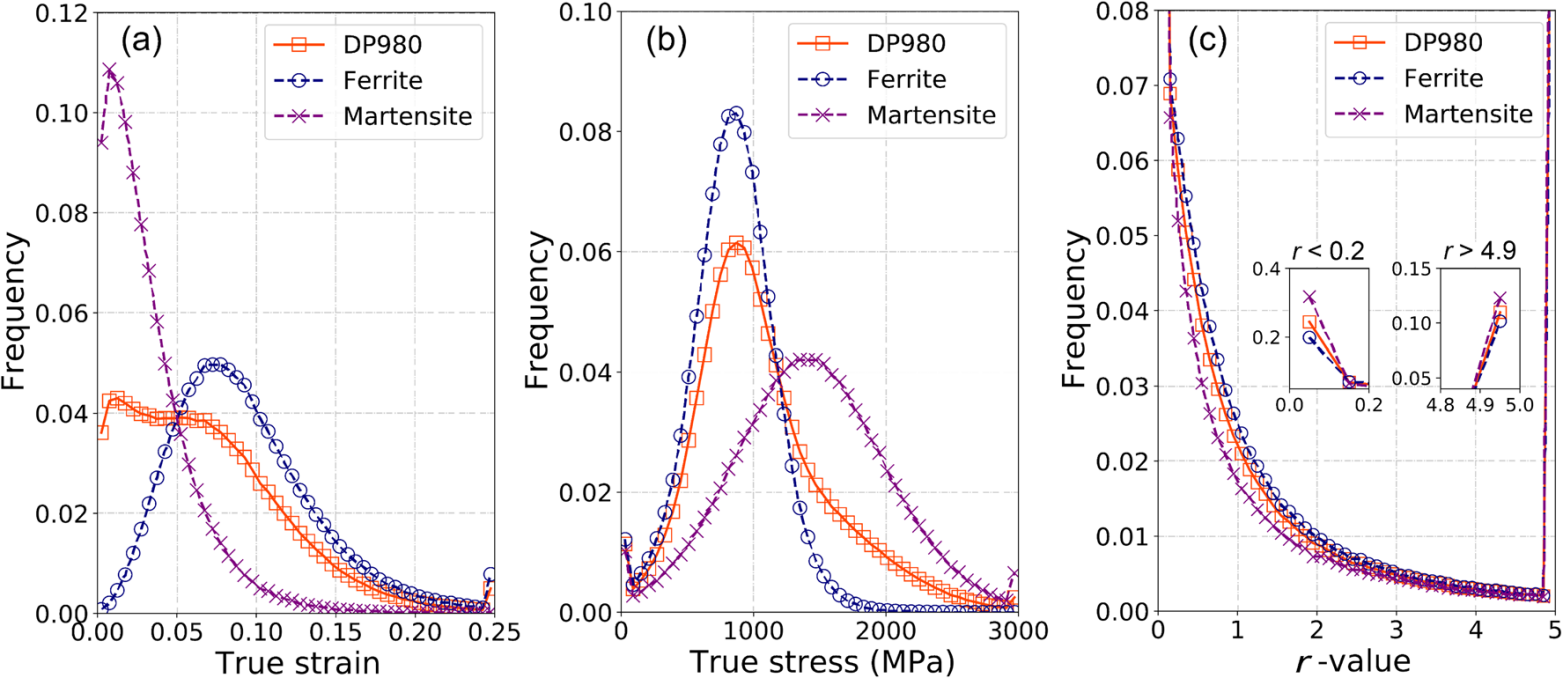
The histograms of the frequency distribution of true strain (bin size 0.005), true stress (bin size 60 MPa), and *r*-value (bin size 0.1) of the DP-RVE and the separated F-RVE and M-RVE. The RVE was subjected to uniaxial tension in RD at the specific plastic work of 60 MPa. The true stress and true strain correspond to the components in tensile direction.

To quantitatively understand the plasticity heterogeneity of the dual-phase microstructure, the histograms of frequency distribution of true strain, true stress, and *r*-value (shown in Figure 10) were further plotted in Fig. 11. It is noted that we did not distinguish the frequencies of strains above 0.5, stresses above 3000 MPa, and *r*-values above 5. For example, as shown in Fig. 11a, the large frequency at true strain ($\varepsilon$) of 0.25 represents the volume fraction of all material points with $\varepsilon \ge 0.25$; in contrast, the frequencies at $\varepsilon <0.25$ were calculated using a bin size of 0.005. As shown in Fig. 11a, the strain frequency distribution of the ferrite essentially follows a Gaussian normal distribution with the median of ~ 0.07, whereas that of the martensite is more like a log distribution with the mode of ~ 0.01. It indicates that the ferrite phase, as expected, accommodates most applied deformation and has more homogeneous deformation than the martensite. Li et al.^17^ demonstrated that the statistical strain distribution of a single-phase material, either ferrite or martensite, follows the normal distribution. The log distribution of the statistical strain of the martensite in the dual-phase structure is inferred because of the strongly non-uniform deformation partition between the phases. Because of the different strain distributions of the individual phases, the DP-RVE (i.e., the actual DP980 sheet) exhibits a non-probability distribution of strain, suggesting a statically non-uniform deformation in the steel sheet. On the contrary, the stress frequency distributions of both phases manifest as the typical Gaussian distribution, as shown in Fig. 11b. This, on the one hand, demonstrates enough number of material points (Fourier grids) and grains (orientations) in the highly-resolved RVE for representing the dual-phase microstructure; on the other hand, it implies the statistically uniform stress distribution of the individual phases. In contrast, the standard deviation of the martensite is obviously larger than that of the ferrite. It implies a more uniform stress distribution in the ferrite. As anticipated, the median stress (~ 1400 MPa) of the martensite is much larger than that (~ 825 MPa) of the ferrite. Besides, unlike the strain frequency distribution, the stress distribution of the DP-RVE is well bell-shaped.

The frequency distribution of *r*-value is rather different from those of strain and stress. For the martensite phase, as shown in Fig. 11c, there are more than 30% material points with *r*-value close to zero and ~ 13% with *r*-value above 5.0, and these values for the ferrite is ~ 20% and ~ 11%, respectively. Apart from these two extreme sides, the *r*-value frequency distributions of the ferrite and martensite phases are greatly similar; both manifest a power-law distribution. These features solidly imply a significant microscopic deformation anisotropy of the DP980 sheet, although its overall normal anisotropy (as shown in Fig. 8) is not very strong. Besides, Fig. 11c displays that the statistical *r*-value of the martensite is noticeably smaller than that of the ferrite. This phenomenon coincides with the initial crystallographic textures shown in Fig. 4 of the two phases, i.e., the martensite’s texture contains a considerable intensity of *α-*fiber, which weakens *r*-value of the steel. Besides, the more inhomogeneous deformation in the martensite might also account for its smaller *r*-value.

It is noted that RVEs tensile tested along other directions exhibit similar results as those presented in Figs. 10 and 11. In summary, these results demonstrate that the CP-based VL with the highly-resolved RVE correlates adequate micro-mechanisms and deformation heterogeneity of the dual-phase microstructure to the macroscopic plastic anisotropy of the DP980 sheet.

### Factors affecting the plastic anisotropy of the DP980 sheet

Unlike the FCC structure of the austenitic steel and aluminum alloy, the BCC structure of the ferritic and martensitic steels has no single family of high-density slip planes, which makes BCC metals generally have more complex plastic deformation than the FCC metals. Many researchers reported the good agreement between the predicted and measured *r*-values for FCC materials such as aluminum and austenitic steel, but poor for BCC carbon steel, ferritic stainless steel, and dual-phase steel^41–43^. Compared with the results obtained from mean-field CP simulations^43,44^, the results predicted by the proposed VL show a good agreement with the experimental data, as shown in Figs. 8 and 9. Whereas, it is also noticed that a relatively large discrepancy between the simulated and experimental *r*-values exists in the DD and TD. To further understand the factors affecting the angular evolution of plastic anisotropy of the dual-phase steel, full-field CP simulations of uniaxial tension along RD, DD, and TD were carried out for single-phase ferritic and martensitic RVEs, respectively; another set of simulations of the dual-phase RVE assuming the same self and latent hardening effect [i.e., $q=1$ in Eq. (4)] were also performed. The comparison among the simulation results, in terms of *r*-values, the average *r*-value, and the planar anisotropy $\Delta r$, were presented in Figure S2 and Table S2 in the supplementary file.

As shown in Table S2, all the simulations predicted larger $\bar{r }$ and $\Delta r$ than experiments; the original dual-phase RVE yields the most close $\bar{r }$ and $\Delta r$ with experiments, and the RVE with the single martensite phase predicts the largest *r*-value. It implies that the dual-phase microstructure reduces the plastic anisotropy. As Rollett and Wright^45^ and Choi et al.^46^ noted, the plastic heterogeneity occurring in a multiphase structure with a soft matrix and hard phases, leads to not only varying magnitudes of strain, but also varying local lattice rotations, all of which tend to disperse texture and reduce anisotropy. It gives one clue to explain the deviation of the simulated *r*-values shown in Fig. 8: although a full-field CP modeling was adopted in this work, the interaction between the two constituent phases, which was enforced by the geometrical constraint among Fourier grids due to deformation compatibility, was much simplified to some extent, especially they have large strength difference and similar volume fraction. On the other hand, the capturing of plastic heterogeneity should be improved by using more advanced CP constitutive theory. A possible way to improve the result is using a physically-based nonlocal crystal plasticity model considering geometrically necessary dislocation enhancement, which can describe a more realistic interaction among heterogeneous microstructures^47^. Besides, the role that crystallography plays at the phase interface has not yet been addressed experimentally and theoretically to much extent.

Another factor affecting the simulation results is the latent hardening matrix (or interaction matrix) in Eq. (4). In this work, the latent hardening matrix was referred to the scheme of Peirce et al.^30^, that the hardening ratio $q=1.0$ for coplanar slip systems and 1.4 for all non-coplanar slip systems. This scheme is pure phenomenological and simplified^48,49^. As a comparison, simulations with $q=1.0$ for all non-coplanar slip systems (the most simplified case) were also carried out. The results presented in Fig. S2 and Table S2 demonstrate that the CP simulations assuming an identical strengthening effect of self-hardening and latent hardening yielded both the larger $\bar{r }$ and $\Delta r$ than the original simulations. With a well-defined interaction matrix considering the different strengthening effects of self-interaction and various collinear and coplanar interactions, one can expect a better performance of CP simulations in capturing the anisotropy of BCC metals. However, the actual latent hardening matrix for a given material is still an open question^50^.

From the aspect of experiments, the mechanical data presented in Figs. 5, 6, 8, and 9 are the macroscopic and bulk mechanical responses of innumerable grains. Although the microstructural RVE was built based upon the EBSD data (as shown in Fig. 4) containing approximately 2000 grains, whereas the EBSD data only represents the crystallographic texture in a local region on the RD-TD plane. Moreover, the thermomechanical operations experienced by the as-received steel generally produce materials with non-uniform spatial distribution of texture. Indeed, texture gradients of both $\gamma$ and $\alpha$ texture fibers through thickness were commonly reported in cold-rolled and hot-rolled steel sheets^43,51^. As pointed out by Kodukula et al.^43^, one of the key reasons for the large discrepancy between measured and calculated *r*-values of a BCC steel is the strong texture gradient through thickness. Increasing the ratio of $\gamma -$ fiber to $\alpha -$ fiber can improve the *r*-value of BCC steel^52^, and this ratio was reported to decrease from the surface to the center of steel sheets^53^. Figure S3 shows the RD and ND IPFs for the microtextures of studied steel characterized on the RD-TD surface and the thickness center, respectively. It shows that the $\gamma -$ fiber is significantly intensified in the center. The RVE used in the VL was built without considering texture gradients, and it only incorporated the microtexture data characterized in the center of the steel, which might have the largest intensity of $\gamma -$ fiber. This could also be a factor resulting in the discrepancy between the simulated and experimental results.

## Summary

A virtual laboratory (VL) based on full-field crystal plasticity (CP) modeling was presented to investigate mechanical anisotropy and to predict yield surfaces of multiphase metals. The VL consists of four modules, including a CP constitutive model, a highly-resolved representative volume element of multiphase microstructure, an inverse simulation procedure based on global optimization for identifying the CP parameters of the constituent phases, and a local optimization scheme for calibrating yield functions through a large number of simulated stress points.

Elaborate mechanical experiments and microstructure characterizations were carried out for modeling setup as well as validation of the CP parameters and of the calibrated yield functions. Both the yield loci generated by the VL and predicted by the calibrated yield functions agree well with the experiments, which were enveloped by yield stress points of uniaxial tensions, pure shear, and biaxial tensions with various stress ratios. The deformation and strength anisotropies were finely captured by the VL and the calibrated yield functions. Especially the Hill48 yield function, which is generally incapable of capturing *r*-value and normalized yield stress (${Y}_{\theta }$) simultaneously, presents a satisfactory prediction of both *r*-value and ${Y}_{\theta }$. This is mainly attributed to the optimized parameters identified from the abundant stress points of arbitrary loading conditions generated by the VL.

Plasticity heterogeneities on grain level were investigated based on the dual-phase RVE, intentionally separated single-phase ferrite and martensite RVEs. It demonstrates that the CP-based VL with highly-resolved RVEs correlates adequately micro-mechanisms and deformation heterogeneity of the multiphase microstructure to the macroscopic plastic anisotropy of the material.

According to the analysis of factors affecting the plastic anisotropy of DP980, the performance of the VL could be improved by introducing a physically-based nonlocal CP model, the role of crystallography plays at the phase interface, a well-defined interaction matrix, and texture gradient through thickness.

## Experiments

### Microstructural characterizations

The initial microstructure and texture of the as-received sheet were characterized by the SEM electron-backscattered diffraction (EBSD) system, i.e., the VEGA 3 XMU (LaB6) field emission SEM (TESCAN) equipped with an Oxford/Nordlys EBSD detector. The SEM-EBSD system was operated at acceleration voltage of 20 kV and working distance of 20 mm; it scanned the area with size of 100 μm × 100 μm and step size of 0.25 μm, i.e., collected 160,000 data points totally. The data was processed by the AZtec® system (version 2.1, Oxford Inst.) and reproduced with the open-source MATLAB® (version R2018b) toolbox MTEX^38^.

### Mechanical tests

To obtain the parameters of the CP constitutive model and to validate the yield functions determined by the full-field CP-based VL, room-temperature mechanical tests, including uniaxial tension, pure shear, and biaxial tension, were conducted for the as-received sheet. The geometric shape and dimension of these specimens are illustrated in Fig. S1a in the Supplementary. The specimens were machined via electrical discharge machining; for the biaxial tensile specimens, seven slits were fabricated on each arm by laser cutting to reduce the geometric constraint on the deformation zone^50^.

Uniaxial tensile tests and pure shear tests were carried out quasi-statically on an electronic testing machine (Instron Model 8080 with a load cell of 100kN capacity) equipped with a commercial digital image correlation (DIC) system (ARAMIS), as shown in Fig. S1b, at constant crosshead speeds of 3 mm/min and 2 mm/min, respectively. Uniaxial tensile tests were carried out in the directions of every 15° from RD to TD, and five experiments were carried out for each direction to obtain the repeatable experimental data. The biaxial tensile tests were carried out along four loading paths (with stress ratios ${\sigma }_{11}:{\sigma }_{22}=1:1, 2:1, 1:2,$ and $4:3$) on a biaxial tensile testing machine (MTS BIA5105) as shown in Fig. S1c at a constant equivalent loading rate 0.49 kN/s. Two load cells were employed to measure the tensile force along RD and TD in real-time.

To facilitate the full-field strain measurements of the DIC system, all the specimens were uniformly sprayed with random speckle patterns prior to the tests. The three-dimensional position change of the speckles was photographed by dual high-resolution cameras at a frequency of 2 Hz; the strain field of the deformed specimens then was computed by the commercial DIC software GOM. The photographing was automatically synchronized with the load cell signal by the DIC data acquisition system.

## Supplementary Information

Supplementary Information.
