# Supplementary material for: A virtual laboratory based on full-field crystal plasticity simulation to characterize the multiscale mechanical properties of AHSS
Source: Sci Rep. 2022 Mar 23;12:5054. doi: 10.1038/s41598-022-09045-8 (PMC8943098; doi:10.1038/s41598-022-09045-8)
Supplement: Supplementary file 1 — Supplementary Information. [file 41598_2022_9045_MOESM1_ESM.docx]

**Supplementary Information**

# A virtual laboratory based on full-field crystal plasticity simulation to characterize the multiscale mechanical properties of AHSS

Hongyue Ma^1,2^, Yangqi Li^1,2^, Haiming Zhang^1,2^*, Qian Li^3^, Fei Chen^1,2^, Zhenshan Cui^1,2^

*1. School of Materials Science and Engineering, Shanghai Jiao Tong University, 800 Dongchuan Road, Shanghai 200240, China*

*2. Institute of Forming Technology & Equipment, Shanghai Jiao Tong University, 1954
Huashan Road, Shanghai, 200030, PR China*

*3. Institute of Shanghai Aircraft Design & Research of Commercial Aircraft Corporation of China, Jinke Road, Shanghai, 201210, PR China*

**Table S1** Chemical composition of the cold-rolled and annealing DP980 steel (wt.%)

| C | Mn | Si | P | S | Al | Fe |
| --- | --- | --- | --- | --- | --- | --- |
| 0.15 | 2.73 | 1.56 | 0.029 | 0.025 | 0.033 | Balance |


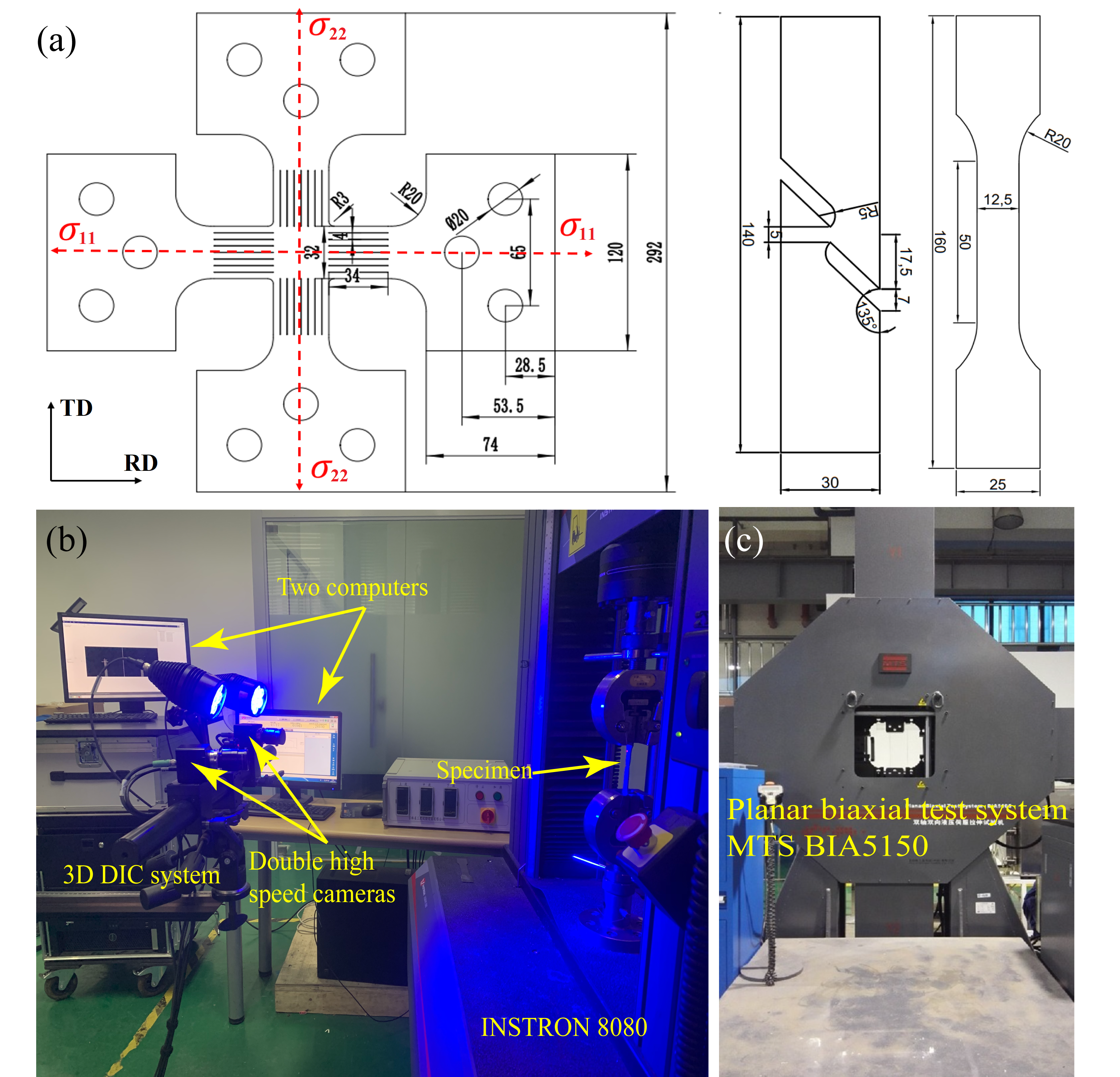


**Figure S1.** (a) The geometric shapes and dimensions of the specimens designed for biaxial tensile, pure shear, and uniaxial tensile tests; $\sigma_{11}$ and $\sigma_{22}$ denote the normal stress in the rolling direction (RD) and transverse direction (TD). The equipments used in the experiments are (b) the universal testing machine of INSTRON 8080 and (c) the planar biaxial test system MTS BIA5105.


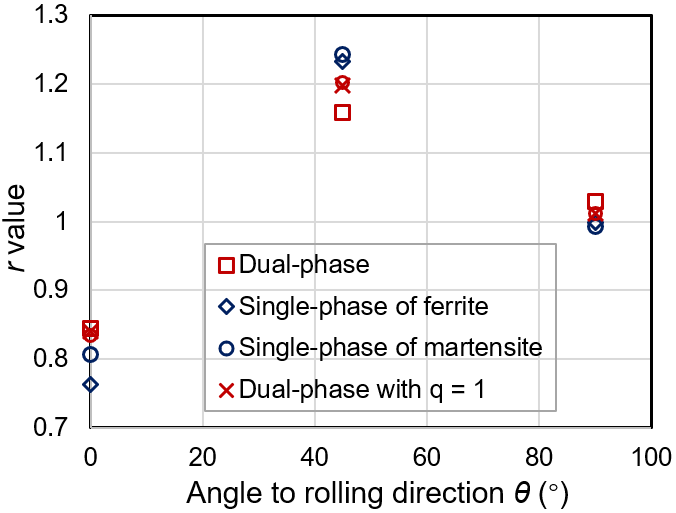


**Figure S2**. $r-$value versus $\theta$ of different RVEs at deformation stage with the specific plastic work of 8 MPa.


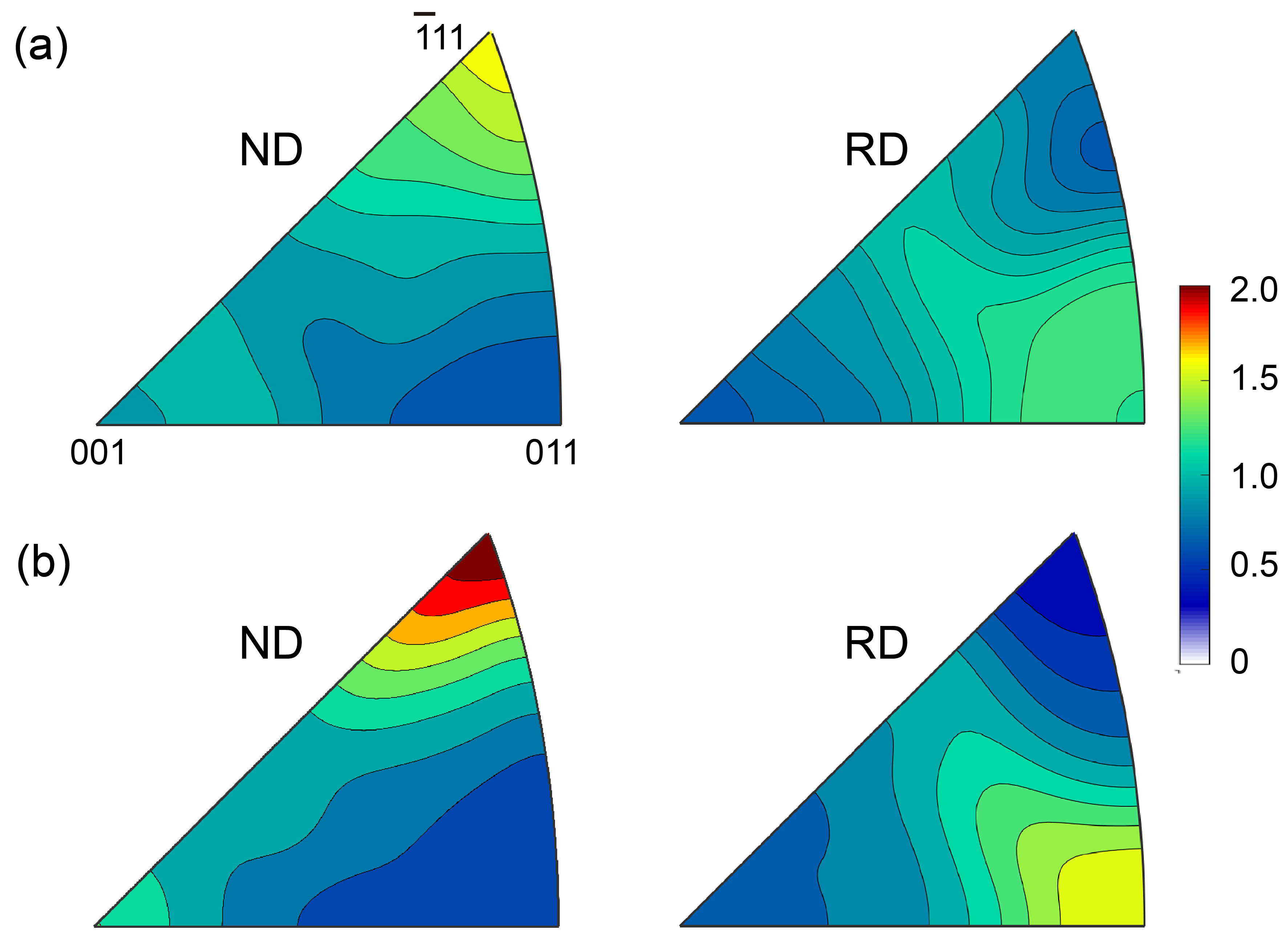


**Figure S3**. The ND and RD IPF maps of the as-received DP980 sheet steel characterized on the RD-TD plane (a) at the surface and (b) at the thickness center. The maps was generated by the MATLAB® (version R2018b) open-source toolbox MTEX ^[38]^.

**Table S2**. Comparation of the average $r-$value and the planar anisotropy between the experiments results and simulation ones.

|  | Experiments | Dual-phase | Single-phase of ferrite | Single-phase of martensite | Dual-phase with $q=1.0$ |
| --- | --- | --- | --- | --- | --- |
| $\bar{r}$ | 0.941 | 1.047 | 1.057 | 1.071 | 1.063 |
| $\Delta r$ | 0.380 | 0.443 | 0.702 | 0.685 | 0.560 |

### Yield functions and the analytical expressions of r-value

The $r-$value $r_{\theta}$ (where $\theta$ is the inclined angle of the uniaxial tensile specimen with respect to the RD) is defined as the ratio of the plastic strain rates associated to width (inclined at the angle $\theta$+90° with respect to the RD), $\dot{\varepsilon}_{\theta+90}$, and thickness, $\dot{\varepsilon}_{33}$

$r_{\theta}=\frac{\dot{\varepsilon}_{\theta+90}}{\dot{\varepsilon}_{33}}$ (1)

If the associated flow rule is used, the above equation can be rewritten in terms of the stress components as

$r_{\theta}=-\frac{\frac{\partial\bar{\sigma}}{\partial\sigma_{11}}\sin^{2}\theta+\frac{\partial\bar{\sigma}}{\partial\sigma_{22}}\cos^{2}\theta-\frac{\partial\bar{\sigma}}{\partial\sigma_{12}}\sin\theta\cos\theta}{\frac{\partial\bar{\sigma}}{\partial\sigma_{11}}+\frac{\partial\bar{\sigma}}{\partial\sigma_{22}}}$ (2)

Where $\bar{\sigma}$ is the effective stress defined by a specific yield function, and $\sigma_{11}$, $\sigma_{22}$, and $\sigma_{12}$ are the in-plane stress components. More details about the derivation can be referred to the work of Banabic ^3^.

### The quadratic Hill48 yield function

The Hill48 yield criterion ^5^ is a typical quadratic yield function; its effective stress is written as:

$\bar{\sigma}=F\left( \sigma_{22}-\sigma_{33} \right)^{2}+G\left( \sigma_{33}-\sigma_{11} \right)^{2}+H\left( \sigma_{11}-\sigma_{22} \right)^{2}+2L{\sigma_{23}}^{2}+2M{\sigma_{31}}^{2}+2N{\sigma_{12}}^{2}$ (3)

where $F,G,H,L,M$, and $N$ are the anisotropic parameters. The expression of $\frac{\partial\bar{\sigma}}{\partial\sigma_{ij}}$ for this function is straightforward, and $r_{\theta}$ is written as

$r_{\theta}=-\frac{G\cos^{2}\theta\sin^{2}\theta+F\cos^{2}\theta\sin^{2}\theta-H\cos^{2}2\theta-\frac{1}{2}N\sin^{2}2\theta}{G\cos^{2}\theta+F\sin^{2}\theta}$ (4)

### The Yld91 yield function

The effective stress of then Yld91 yield criterion ^9^ is:

$\bar{\sigma}=\frac{\sqrt{3I_{2}}}{2^{1/m}}\left( \left| \emptyset_{1} \right|^{m}+\left| \emptyset_{2} \right|^{m}+\left| \emptyset_{3} \right|^{m} \right)^{1/m}$ (5)

with $\emptyset_{1}=2\cos\left( \frac{2\alpha+\pi}{6} \right)$，$\emptyset_{2}=2\cos\left( \frac{2\alpha+3\pi}{6} \right)$, $\emptyset_{3}=2\cos\left( \frac{2\alpha+5\pi}{6} \right)$ and $\alpha=\arccos\left( \frac{I_{3}}{I_{2}^{3/2}} \right)$. $I_{2}$ and $I_{3}$ are the second and the third invariants of stress tensor, respectively. For the anisotropic case, $I_{2}$ and $I_{3}$ become:

$I_{2}=\frac{\left( fF \right)^{2}+\left( gG \right)^{2}+\left( hH \right)^{2}}{3}+\frac{\left( aA-cC \right)^{2}+\left( cC-bB \right)^{2}+\left( bB-aA \right)^{2}}{54}$ (6)

$I_{3}=\frac{\left( cC-bB \right)\left( aA-cC \right)\left( bB-aA \right)}{54}+\left( fgh \right)\left( FGH \right)-\frac{\left( cC-bB \right)\left( fF \right)^{2}+\left( aA-cC \right)\left( gG \right)^{2}+\left( bB-aA \right)\left( hH \right)^{2}}{6}$ (7)

where $A=\sigma_{22}-\sigma_{33}$, $B=\sigma_{33}-\sigma_{11}$, $C=\sigma_{11}-\sigma_{22}$, $F=\sigma_{23}$, $G=\sigma_{31}$, and $H=\sigma_{12}$. $a,b,c,f,g,h,$ and $m$ are material parameters.

By means of chain rule of derivative, $\frac{\partial\bar{\sigma}}{\partial\sigma_{ij}}$ is explained as follows,

$\frac{\partial\bar{\sigma}}{\partial\sigma_{ij}}=\frac{\partial\bar{\sigma}}{\partial I_{2}}\frac{\partial I_{2}}{\partial\sigma_{ij}}+\frac{\partial\bar{\sigma}}{\partial\alpha}\frac{\partial\alpha}{\partial\sigma_{ij}}$ and $\frac{\partial\bar{\sigma}}{\partial\alpha}=\frac{\partial\bar{\sigma}}{\partial\emptyset_{i}}\frac{\partial\emptyset_{i}}{\partial\alpha}$ (8)

The last term follows the Einstein summation convention. By means of the direct mathematical operations, the components in Eq. 5 are obtained as

$\frac{\partial\bar{\sigma}}{\partial I_{2}}=\frac{\bar{\sigma}}{2I_{2}}$ (9)

$\frac{\partial\bar{\sigma}}{\partial\emptyset_{i}}=\frac{\sqrt{3I_{2}}}{2^{1/m}}\left( \left| \emptyset_{1} \right|^{m}+\left| \emptyset_{2} \right|^{m}+\left| \emptyset_{3} \right|^{m} \right)^{1/m-1}\left| \emptyset_{i} \right|^{m-2}\emptyset_{i}$ (10)

$\frac{\partial\alpha}{\partial\sigma_{ij}}=\frac{\partial\alpha}{\partial I_{2}}\frac{\partial I_{2}}{\partial\sigma_{ij}}+\frac{\partial\alpha}{\partial I_{3}}\frac{\partial I_{3}}{\partial\sigma_{ij}}$, with $\frac{\partial\alpha}{\partial I_{2}}=\frac{-3I_{3}}{2I_{2}^{5/2}\sqrt{1-{I_{3}^{2}}/{I_{2}^{3}}}}$ and$\frac{\partial\alpha}{\partial I_{3}}=-\frac{2I_{2}}{3I_{3}}\frac{\partial\alpha}{\partial I_{2}}$ (11)

While $\frac{\partial I_{2}}{\partial\sigma_{ij}}$, $\frac{\partial I_{3}}{\partial\sigma_{ij}}$, and $\frac{\partial\emptyset_{i}}{\partial\alpha}$ are easily accessible. In the end, a complete expression of $\frac{\partial\bar{\sigma}}{\partial\sigma_{ij}}$ reads,

$\frac{\partial\bar{\sigma}}{\partial\sigma_{ij}}=\left( \frac{\partial\bar{\sigma}}{\partial I_{2}}+\frac{\partial\bar{\sigma}}{\partial\alpha}\frac{\partial\alpha}{\partial I_{2}} \right)\frac{\partial I_{2}}{\partial\sigma_{ij}}+\frac{\partial\bar{\sigma}}{\partial\alpha}\frac{\partial\alpha}{\partial I_{3}}\frac{\partial I_{3}}{\partial\sigma_{ij}},$ (12)

Substitute the aforementioned components into Eq. 8, one can obtain the specific expressions of $\frac{\partial\bar{\sigma}}{\partial\sigma_{11}}$, $\frac{\partial\bar{\sigma}}{\partial\sigma_{22}}$, and $\frac{\partial\bar{\sigma}}{\partial\sigma_{12}}$. $r_{\theta}$ then can be derived based on Eq. 2.

### The Yld2004-18p yield function

The Yld2004-18p anisotropic yield function proposed by Barlat *et al.* ^12^ is one of the most widely used advanced yield functions; its effective stress is defined as follows,

$\bar{\sigma}=\left( \frac{1}{4}\emptyset\right)^{\frac{1}{m}}$ (13)

where $m$ is the homogeneous exponent related to the crystal structure of materials. $\emptyset$ is expressed as follows:

$$\emptyset=\left| {s^{'}}_{1}-{s^{''}}_{1} \right|^{m}+\left| {s^{'}}_{1}-{s^{''}}_{2} \right|^{m}+\left| {s^{'}}_{1}-{s^{''}}_{3} \right|^{m}+\left| {s^{'}}_{2}-{s^{''}}_{1} \right|^{m}+\left| {s^{'}}_{2}-{s^{''}}_{2} \right|^{m}$$

$+\left| {s^{'}}_{2}-{s''}_{3} \right|^{m}+\left| {s^{'}}_{3}-{s''}_{1} \right|^{m}+\left| {s^{'}}_{3}-{s''}_{2} \right|^{m}+\left| {s^{'}}_{3}-{s''}_{3} \right|^{m}$ (14)

where ${s^{'}}_{i}$ and ${s''}_{i}$ $\left( i=1, 2, 3 \right)$ are the principal values of tensors $\mathbf{S}^{\mathbf{'}}$ and $\mathbf{S}\boldsymbol{''}$, which are defined by liner transformations of deviatoric part of Cauchy stress tensor $\boldsymbol{\sigma'}$:

$\mathbf{S}^{\boldsymbol{'}}\boldsymbol{=C:}\boldsymbol{\sigma'}\boldsymbol{,}\mathbf{S}\boldsymbol{’’=D:}\boldsymbol{\sigma'}$ (15)

The fourth-order tensors $\boldsymbol{C}$ and $\boldsymbol{D}$ characterize the anisotropy of the material, and each contains nine material parameters as follows (in Voigt notation).

$\boldsymbol{C}=\left[ \begin{matrix} 0 & -c_{12} & -c_{13} & 0 & 0 & 0 \\ -c_{21} & 0 & -c_{23} & 0 & 0 & 0 \\ -c_{31} & -c_{32} & 0 & 0 & 0 & 0 \\ 0 & 0 & 0 & c_{44} & 0 & 0 \\ 0 & 0 & 0 & 0 & c_{55} & 0 \\ 0 & 0 & 0 & 0 & 0 & c_{66} \end{matrix} \right]$ (16)

$\boldsymbol{D}=\left[ \begin{matrix} 0 & -d_{12} & -d_{13} & 0 & 0 & 0 \\ -d_{21} & 0 & -d_{23} & 0 & 0 & 0 \\ -d_{31} & -d_{32} & 0 & 0 & 0 & 0 \\ 0 & 0 & 0 & d_{44} & 0 & 0 \\ 0 & 0 & 0 & 0 & d_{55} & 0 \\ 0 & 0 & 0 & 0 & 0 & d_{66} \end{matrix} \right]$ (17)

The identified coefficients include the elements in the tensors $\boldsymbol{C}$ and $\boldsymbol{D}$ and the exponent *m*. This yield function contains 18 anisotropic parameters, among which $c_{55}$, $c_{66}$, $d_{55}$ and $d_{66}$ are used to characterize the out-of-plane anisotropies, and the other 14 parameters describe the in-plane properties of the sheet. Note that the Yld2004-18p reduces to the Yld91 yield function when $\boldsymbol{C=D}$.

By means of chain rule of derivative, $\frac{\partial\bar{\sigma}}{\partial\sigma_{ij}}=\frac{\partial\bar{\sigma}}{\partial\emptyset}\frac{\partial\emptyset}{\partial\sigma_{ij}}$, and

$\frac{\partial\bar{\sigma}}{\partial\emptyset}=\frac{1}{4m}\cdot\left( \frac{1}{4}\emptyset\right)^{\frac{1}{m}-1}$ (18)

$\frac{\partial\emptyset}{\partial\sigma_{ij}}=\frac{\partial\emptyset}{\partial s_{k}^{'}}\frac{\partial s_{k}^{'}}{\partial\sigma_{ij}}+\frac{\partial\emptyset}{\partial s_{k}^{''}}\frac{\partial s_{k}^{''}}{\partial\sigma_{ij}}$ (19)

As illustrated in Eq. 15, the principal stresses $s_{k}^{'}$ ($k=1, 2, 3$) and $s_{k}^{''}$ have the same form. In the following, we focus the derivation associated with $s_{k}^{'}$. First, the expression of $s_{k}^{'}$ reads,

$s_{k}^{'}=\frac{I_{1}}{3}$+$\frac{2}{3}\sqrt{I_{1}^{2}-3I_{2}}\cos\left[ \alpha+\frac{2\pi}{3}k \right], k=1, 2, 3$ (20)

with

$\alpha= \arccos\left( \frac{2I_{1}^{3}-9I_{1}I_{2}+27I_{3}}{2\left( I_{1}^{2}-3I_{2} \right)^{3/2}} \right)-\frac{2\pi}{3}$ (21)

where $I_{1}, I_{2}$, and $I_{3}$ are the stress invariants of the stress tensor $\mathbf{S}^{\mathbf{'}}$. Thus, $s_{k}^{'}$ is a function of $I_{1}, I_{2}$, and $I_{3}.$ Therefore

$\frac{\partial s_{k}^{'}}{\partial\sigma_{ij}}=\frac{\partial s_{k}^{'}}{\partial I_{m}}\frac{\partial I_{m}}{\partial\sigma_{ij}}$ (22)

where

$\frac{\partial s_{k}^{'}}{\partial I_{1}}=\frac{1}{3}+\frac{2}{3}\left( I_{1}^{2}-3I_{2} \right)^{{-1}/2}I_{1}\cos\left( \alpha+\frac{2k\pi}{3} \right)-\frac{2}{3}\sqrt{I_{1}^{2}-3I_{2}}sin\left( \alpha+\frac{2k\pi}{3} \right)\frac{\partial\alpha}{\partial I_{1}}$ (23)

$\frac{\partial s_{k}^{'}}{\partial I_{2}}=-\left( I_{1}^{2}-3I_{2} \right)^{-1/2}\cos\left( \alpha+\frac{2k\pi}{3} \right)-\frac{2}{3}\sqrt{I_{1}^{2}-3I_{2}}sin\left( \alpha+\frac{2k\pi}{3} \right)\frac{\partial\alpha}{\partial I_{2}}$ (24)

$\frac{\partial s_{k}^{'}}{\partial I_{3}}=-\frac{2}{3}\sqrt{I_{1}^{2}-3I_{2}}sin\left( \alpha+\frac{2k\pi}{3} \right)\frac{\partial\alpha}{\partial I_{3}}$ (25)

Since $\frac{\partial I_{m}}{\partial\sigma_{ij}}$ and $\frac{\partial\alpha}{\partial I_{m}}$ ($m=1,2,3$) can routinely derived. $\frac{\partial\emptyset}{\partial\sigma_{ij}}$ can be obtained through the assembly of the above equations.
